# Working conditions and labour rights coverage of women domestic workers in Peru: a respondent-driven sampling study

**DOI:** 10.1186/s12889-026-28527-2

**Published:** 2026-07-14

**Authors:** Janeth Tenorio-Mucha, Christopher Meaney, David Vera-Tudela, Marcolina Infante Ramirez, Sofia Mauricio Bacilio, María de los Ángeles Ochoa Luján, Adelina Díaz Uriarte, Marta Dolorier, Leddy Mozombite Linares, Ernestina Ochoa Luján, Karina Romero, Sebastian Garcia Cruz, Guillermo Almeida-Huanca, Nathaly Aya Pastrana, Maria Kathia Cardenas, Maria Lazo-Porras, Michael Rotondi, Archna Gupta

**Affiliations:** 1 CRONICAS Centre of Excellence in Chronic Diseases, Universidad Peruana Cayetano Heredia, Av. Armendariz 445 – Miraflores, Lima, Peru; 2 Department of Family and Community Medicine, Faculty of Medicine, University of Toronto, Toronto, Ontario Canada; 3 Universidad Peruana Cayetano Heredia, Lima, Peru; 4 Sintrahol, Sindicato de Trabajadoras y Trabajadores del Hogar de la Región Lima, Lima, Peru; 5 AGTR, Asociación Grupo de Trabajo Redes - Casa Panchita, Lima, Peru; 6 Fentrahogarp, Federación Nacional de Trabajadoras y Trabajadores del Hogar del Perú, Lima, Peru; 7 CCTH, Centro de Capacitación para Trabajadoras del Hogar, Lima, Peru; 8 Fentrahop, Federación Nacional de Trabajadoras y Trabajadores del Hogar del Perú, Lima, Peru; 9 IPROFOTH, Instituto de Promoción y Formación de las Trabajadoras del Hogar, Lima, Peru; 10 IMEK Centro de Investigación en Mercadeo & Desarrollo, Santiago de Cali, Colombia; 11 School of Kinesiology and Health Science, York University, Toronto, Ontario Canada; 12 Upstream Lab, MAP Centre for Urban Health Solutions, Li Ka Shing Knowledge Institute, St. Michael’s Hospital, Unity Health Toronto, Toronto, Ontario Canada; 13 Department of Family and Community Medicine, St. Michael’s Hospital, Unity Health Toronto, Toronto, Ontario Canada

**Keywords:** Domestic workers, Working conditions, Informal sector, Income, Labour rights

## Abstract

**Background:**

Worldwide, 81% of domestic workers (DWs) lack labour rights due to absent labour-protection laws or a lack of enforcement of existing laws. Paid domestic work is a vital source of employment for women in Latin America, yet most DWs continue to experience precarious working conditions and limited access to labour rights. This study describes working conditions and labour-rights coverage among women DWs in Peru and examines variation by employment status.

**Methods:**

We conducted a cross-sectional survey using respondent-driven sampling (RDS) to recruit 456 women DWs in Peru, across the regions of Lima, La Libertad, and Piura between 2023 and 2024. Eligible participants were women over 14 years whose primary income was derived from domestic work, including full-time, part-time, live-in, or live-out employment in the employer’s household, as well as single- and multi-employer arrangements. We collected detailed sociodemographic, employment, and labour rights data. We estimated unweighted and RDS-II weighted proportions and stratified by employment status (formal versus informal). Formal employment was defined as having a written contract—simple, notarized, arranged through an outsourcing agency, or legally registered—whereas informal employment included only verbal agreements or no contract at all.

**Results:**

In our sample, most women DWs were aged 19 to 45 years and were single. Most were live-out, worked under informal employment, for a single employer, and worked between 7 and 9 h/day. Overall, coverage of labour rights was low, particularly for receiving payslips (2.8%) and severance pay (3.3%). DWs with informal employment were less likely to receive employer-paid social security (23.4%, 95% CI: 0.0-48.2 vs. 67.8%, 95% CI:19.0-100.0), retirement pension (7.0%, 95% CI: 1.3–12.6 vs. 20.9%, 95% CI:4.4–37.4), and paid vacations (24.0%, 95% CI: 16.9–31.0 vs. 41.9%, 95% CI: 20.0-63.8) than DWs with formal employment. The most common barriers to accessing labour rights include a lack of employer-provided coverage, a preference for other insurance schemes, and part-time work or having multiple employers.

**Conclusion:**

Despite legislative advances, DWs in Peru remain largely excluded from meaningful labour protections. Strengthening enforcement, improving employer accountability, and clarifying rights for part-time and multi-employer arrangements are critical to advancing equity and social protection for DWs.

**Supplementary Information:**

The online version contains supplementary material available at https://doi.org/10.1186/s12889-026-28527-2.

## Background

Paid domestic work is a vital employment sector, comprising 2.3% of jobs worldwide and approximately 5% in Latin America [1]. Women overwhelmingly make up the domestic workforce, performing tasks such as caregiving, cleaning, and household management. The persistent societal perception that domestic work is not “real work” influences how labour rights are extended or denied to those who work in this sector [2]. Labour rights and social protections typically encompass entitlements such as standard working hours, weekly rest days, paid annual leave, minimum wage protections, parental leave, and paid parental leave benefits, among others [1].

In 2011, the International Labor Organization (ILO) adopted Convention 189 [3], aiming to ensure decent working conditions, fair treatment, and formal recognition of domestic work.

Consequently, many countries have extended, adapted or developed national labour laws to recognize and protect domestic worker (DW) rights [1]. However, the existence of a legal framework does not guarantee effective coverage; in practice, many DWs continue to face barriers to accessing the labour rights and social protections they are entitled to. Globally, many DWs are excluded from regulations on working hours, minimum wage, and social insurance coverage compared with other working sectors [4–6]. During the COVID-19 pandemic, DWs were often ineligible for unemployment or disability benefits [7, 8]. Following pregnancy, some return to work prematurely due to financial hardship and lack of maternity benefits [9].

The domestic work sector is highly diverse, and changes in working conditions over time have led to fragmented legal recognition and limited access to labour rights. DWs include individuals employed full-time by a single household, working part-time for multiple employers, live-in workers who live and sleep in their employers’ household, agency-based employees, and others engaged via digital platforms such us apps or social media post. In Latin America, domestic work is highly feminized and the average age of paid DWs increased from 34.5 to 42.2 years over the past two decades, with 73.8% now concentrated in the 30–64 age group [10]. Employment conditions have also changed; live-in domestic work declined from 22.6% in 2000 to 7.3% today [10]. Workweeks more than 45 h fell from 53.4% to 37.8%, but short part-time schedules have increased [11].

Worldwide, 81% of DWs work within the informal economy, leaving many without effective access to labour rights and social protection due to limited legal coverage or weak enforcement of existing regulations [11]. In many countries in the Middle East, Africa, and Asia, social protection coverage is limited due to a lack of legal frameworks. However, in Europe, Central Asia and the Americas, it is due to a lack of enforcement of existing laws [11].

In Peru, as well as in other countries of the Latin American region, labour informality is highly prevalent, with 55% of workers employed outside formal agreements [12]. This informality disproportionately impacts women, since 73% of employed women work in informal jobs [12]. Pre-existing gender norms, unequal care burdens, low socioeconomic status, limited education, minority ethnic or linguistic backgrounds, and exposure to gender-based violence further constrain women’s access to formal employment.

The high informalization of domestic work in Peru is rooted in colonial systems that relied on enslaved, Indigenous, and Afro-descendant women for unpaid or coerced household labour [13]. This institutionalized the idea that household and care work were low-status, inherently female, and undeserving of formal recognition or remuneration [14]. Contemporary patterns where DWs are predominantly women from marginalized groups reflect enduring colonial legacies. The high level of informality is, however, is a multifactorial problem: at the policy level, enforcement capacity is often limited, and the fact that domestic work takes place within private households poses significant challenges for monitoring compliance [9]. At the employer level, gaps in knowledge and a lack of commitment to labour obligations are common [7]. Meanwhile, at the employee level, many DWs lack awareness of their rights or the confidence to assert them, often fearing job loss if they do so [9, 15].

Although prior studies have examined this sector, most rely on secondary data that fail to adequately capture the realities of a highly informal and marginalized workforce. This study contributes novel evidence by deploying a survey specifically designed for DWs, using a sampling strategy aiming to target hard-to-reach populations, implementing it across three major Peruvian cities, and exploring reasons for accessing labour rights. As most DWs are women, addressing their labour conditions is also central to reducing gender-based health disparities and advancing women’s rights, making this investigation particularly relevant for the public health field. Our primary objective is to describe the working conditions and labour rights coverage of women DWs in Peru. A secondary objective is to examine how access to these rights varies by employment status (informal versus formal).

### Context

Domestic work in Peru is highly heterogeneous and largely informal, often marked by long working hours, low wages, and limited written contracts or payslips. Simple written agreements with vague specifications about labour rights or without formal legal registration may exist and are widely accepted by both employers and DWs [16]. In 2018, Peru’s government endorsed ILO Convention 189, which triggered a movement in the republic’s own legislature. The DWs’ law - Law 31,047 [17] - was published in October 2020. The regulation (D.S. 009-2021-TR), which is a separate legal instrument that operationalized the law by defining procedures, responsibilities, and enforcement mechanisms was published in April 2021. The law and its regulations guarantee written contracts, an 8-hour workday (48-hour workweeks), paid leave and holidays, bonuses, severance pay, social security affiliation, and protections against discrimination and harassment, while also recognizing the right to unionize. It applies to full-time or part-time workers, whether they reside in the employer’s home, and is complemented by a national digital registry of employment contracts.

## Methods

### Study design and setting

This study is part of a larger mixed-methods study, the ANITA project, which is a participatory-action research study that aimed to examine the working conditions, health status, and access to healthcare for women DWs in Peru. The protocol of the full study has been published previously [18].

This paper corresponds to a cross-sectional survey of DWs in 3 different cities in Peru: Lima (the capital), Piura and La Libertad (located in the Northern coast of Peru). Based on data from the 2020 Household National Survey (ENAHO, in Spanish), 51% of DWs reported living in Lima, whereas the other regions with higher concentrations of DWs were Piura (7%) and La Libertad (6%).

### Research ethics

This study received Research Ethic Board (REB) approval from Universidad Peruana Cayetano Heredia in Peru (#544-48-22 and #286-21-23) and Unity Health Toronto in Canada (REB #23–196). The survey was conducted between September and December 2023 in Lima, and between February and March 2024 in La Libertad and Piura.

### Participants and recruitment

Individuals over the age of 14 who identified as women and whose primary source of income was employment as a DW were considered eligible. We included individuals between 14 and 17, aligning with the National Household Survey definition of income earners [19]. We included participants who work full-time or in variable part-time arrangements (including working only a few hours per day or irregular schedules), live-in or live-out the employers’ household, as well as single household (one employer) and multi-household workers multiple employers). We excluded women who do unpaid domestic work and those whose primary income source was not domestic work.

### Study variables

#### Sociodemographic characteristics

Sociodemographic variables included age, gender identity, marital status, migration status, level of education, self-identified ethnicity, mother tongue, whether they were the head of the household, and, if they were living with their own children, how many children they lived with. We did not collect information on sex assigned at birth, but on gender self-identification. Gender was categorised as cis women and non-cis women, with the latter including trans women and non-binary individuals. Socioeconomic status was assessed by asking about access to basic services, including electricity and water. Migration status was categorized as non-migrant, internal migrants for those who moved within the country, and international migrants for those who moved to Peru from abroad. We also collected information about whether participants belonged to a DW organization, including unions, training groups, and advocacy groups, and how many years they have been working as a DW.

#### Working conditions

Working conditions variables included job modality (live-in or live-out), number of employers (single or multiple), payment frequency (daily, weekly, fortnightly, or monthly), and payment amount. We collected information on tasks that DWs performed, including cleaning, cooking, laundry, childcare, shopping, animal care, elderly care, gardening, and care for people with disabilities. We constructed a composite measure corresponding to the number of tasks a DW self-reported performing.

We used employment status as a stratification variable. This variable was originally collected in six categories and, for analysis, reclassified into two groups: informal employment (no contract or agreement, or a verbal agreement only), and formal employment (a simple written contract, a notarized written contract, a contract with an outsourcing agency, or a written contract registered with the Ministry of Labor).

#### Labour rights

Variables related to labour-rights coverage were based on those stipulated in Law 31,047.

They included knowledge of the law, timely payment, at least one day off per week, overtime payment, double payment for working on holidays, medical leave, employer-paid social security, retirement pension, severance pay, payslips, paid vacations. We also collected information about monthly wage at or above the legal minimum (1025 PEN) (1 PEN = 0.29 USD). Given the heterogeneity in payment frequency among DWs, wages were standardized to different time frames for comparability. The monthly minimum wage (1025 PEN or 297 USD) was converted into equivalent fortnightly (513 PEN or 148 USD), weekly (256 PEN or 74 USD), and daily (45 PEN or 11 USD) wages by dividing the total amount by 15, 7, and 1 day, respectively.

We also explored reasons for accessing or not accessing three key labour rights: written legal contract, retirement pension, and employer-paid social security. Participants who reported not receiving a specific benefit answered questions about barriers to access, while those who reported receiving it answered questions about facilitators of access. Information was collected using a multiple-response format with predefined options developed by the research team. An “Other” option with an open-text field was also included to allow participants to provide responses not captured by the predefined categories. Responses are presented as originally reported, including the open-ended responses provided under “Other”.

### Survey development

The questionnaire was developed in collaboration with the DW co-researchers [18]. The questionnaire is available in Supplementary material 1. For sociodemographic characteristics, we used items from the National Household Survey (ENAHO, in Spanish) and the Demographic and Health Survey (ENDES, in Spanish). For working conditions, we adapted items from the European Working Conditions Survey [20] and from the Peruvian Working Conditions, Safety and Health Survey (CENSOPAS) [21]. For knowledge and labour rights coverage, we included questions related to rights and responsibilities stated in the Law 31,047 [17]. In August 2023, a pilot test was conducted with 12 DW leaders to evaluate the completion time and to assess the clarity and relevance of the questions.

### Data collection

This study used respondent-driven sampling, a network-based recruitment approach that begins with a small number of participants known as seeds and then propagates into recruitment waves. Study investigators and DW co-researchers selected seeds to reflect the diversity of DWs, in terms of age, type of employment, working hours, live-in status, work experience, and membership in DW’s unions. We recruited 12 seeds in Lima, four seeds in La Libertad, and five seeds in Piura. Each participant was limited to three peer referrals (although one participant recruited four) this was intended to encourage equal referral opportunities across all participants. Participants received a dual incentive: 70 PEN or 20 USD upon survey completion and 35 PEN or 10 USD for each referred participant who consented to participate. Each referral coupon included a phone number for referrals to contact recruiters (Supplementary Material 2).

Recruiters verified the coupon codes, conducted a pre-screening phone call to confirm eligibility, and scheduled data collection. All participants provided informed consent. Data collection was conducted in person. In Lima, participants could choose between two university offices and a Domestic Workers Union office. In La Libertad and Piura, data were collected at specified offices located in central areas.

Given the study population and the sensitivity of some questions, all research team members who were in contact with participants were trained in ethics, gender-responsive approaches, psychological first aid, and study procedures. Data collection was conducted by trained women research assistants using tablets and REDCap. After participation, all respondents received printed materials on the Domestic Workers Law, written contract templates, and contact information for support services in cases of domestic or workplace violence.

### Sample size

The sample size calculation was based on the primary outcome of the ANITA study, measuring the number of working hours per week and the hourly wage of DWs. The 2021 National Household Survey [22] reported that women DWs averaged 40.4 workweek (standard deviation:19.1). Using this information, we estimated that 224 individuals were required to be enrolled in our study, such that 95% confidence statements could be estimated with a precision (confidence interval (CI) total width) of 5 h. Given that RDS designs often require sample inflation due to high design effects (up to 10) [23], we applied a conservative design effect of 2.0, resulting in a final sample size 448 DWs [18].

The sample size was determined based on the requirements of a study designed to estimate a single continuous outcome - hours worked - with a specified level of precision. The study was not originally powered to detect differences in working conditions or access to labour rights coverage between formally and informally employed DWs.

### Statistical analysis

Working conditions and labour rights coverage were summarized using counts and unweighted percentages. To account for the RDS design, we also present RDS-II weighted estimates of proportions with 95% confidence intervals [24]. RDS-II weights were calculated as the inverse of each participant’s reported network size and normalized to sum to one. We measured the network size of each participant by asking: “How many domestic workers do you know in the region where you live?”. These weights adjust for unequal recruitment probabilities due to differences in social connectivity.

Additionally, we examined differences in sociodemographic and work-related characteristics and access to labour policies across formal and informal employment status using bootstrap RDS methods. We used the RDS: bootstrap.contingency.table() function from the RDS package with RDS-II weights [25], B = 10 000 bootstrap replicate samples and a single randomly chosen fixed seed for reproducibility. P-values were inestimable if the underlying contingency table contained zero valued cells. Statistical significance was declared if the observed bootstrap p-value was less than 0.05.

## Results

**Fig. 1:**
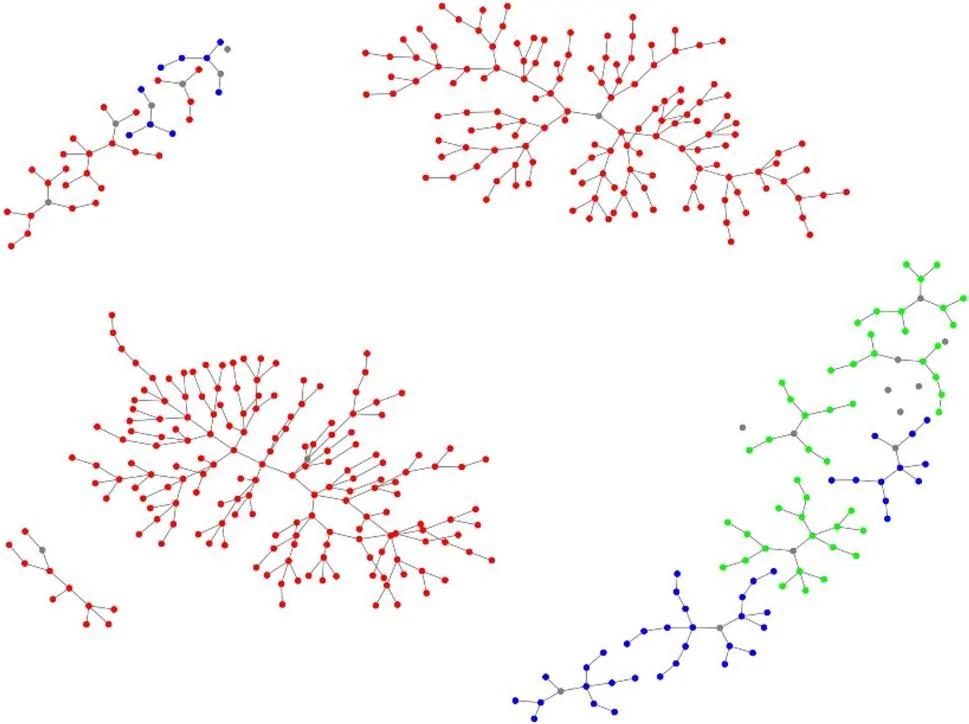
Network diagrams depicting the DW recruitment waves associated with each seeded individual: grey dots represent seeds, red dots represent recruited participants from Lima, blue from Piura, and green from La Libertad

A total of 1368 coupons were distributed in the study. We assessed 489 individuals for study eligibility and excluded 33 during the pre-screening phone call. Reasons for exclusion include occasional domestic work (*n* = 14), work as a janitor or cleaner (*n* = 12), domestic work is not the main source of income (*n* = 2), and unemployment (*n* = 5). We interviewed and analysed data from a total of 456 DWs, including 21 seeds and 435 recruited participants. Figure 1 shows the RDS recruitment network and waves propagated by each seed; the maximum number of waves recruited was 12.

Of the 456 participants, 350 (69.8%) lived and worked in Lima, 51 (17%) in La Libertad, and 55 (13.2%) in Piura. Most participants self-identified as cis women. The majority were between the ages of 19 and 45 (60.0%) and had a secondary education or more (65.4%). Most DWs self-identified as mestizo or with mixed identities (54.8%), 15.1% as Indigenous, 7.7% as Afro Peruvian or Afro descendant, and 22.3% as other which includes white, Andean, Asian descendant or do not know.

Additionally, 4.4% reported an Indigenous language like Quechua, Aimara, or an Amazonian language as their mother tongue. There were 21.3% internal migrants and 6.0% international migrants (Table 1).

**Table 1:** Counts, percentages and 95% confidence intervals describing the socio-demographic and working characteristics of the study participants

Characteristic	Unadjusted estimate		RDS-II adjusted estimate
	*n*	% (95 CI)	% (95 CI)
Age (*n* = 456)			
15–18 years	13	2.9 (1.5–5.5)	4.3 (1.1–7.4)
19–45 years	265	58.1 (52.3–63.7)	60.0 (50.1–70.0)
46–64 years	165	36.2 (30.8–42.0)	32.7 (22.8–42.7)
≥ 65 years	13	2.9 (1.5–5.5)	3.0 (0.0–6.0)
Education (*n* = 364)			
Less than secondary	132	36.3 (30.8–42.1)	34.6 (22.5–46.6)
Secondary or higher	232	63.7 (57.9–69.2)	65.4 (53.4–77.5)
Self-identified ethnicity (*n* = 456)			
Mestizo	233	51.1 (45.3–56.9)	54.8 (45.0–64.7)
Indigenous	86	18.9 (14.7–23.8)	15.1 (5.3–24.9)
Afro Peruvian or Afro descendant	43	9.4 (6.5–13.4)	7.7 (4.7–10.7)
Other	94	20.6 (16.3–25.7)	22.3 (14.8–29.9)
Mother tongue (*n* = 456)			
Spanish	432	94.7 (91.9–96.6)	95.6 (93.9–97.2)
Indigenous	24	5.3 (3.4–8.1)	4.4 (2.8–6.1)
Migration status (*n* = 444)			
Non migrant	326	73.4 (68.1–78.1)	72.7 (61.4–83.9)
Internal migrant	102	23.0 (18.6–28.1)	21.3 (14.0–28.7)
International migrant	16	3.6 (2.0–6.4)	6.0 (0.0–17.3)
Access to potable water (*n* = 456)			
Public network with own meter	224	49.1 (43.6–54.7)	51.4 (41.5–61.4)
Public network with shared meter	170	37.3 (32.0–42.8)	35.1 (26.0–44.3)
Other	62	13.6 (10.2–17.9)	13.4 (7.5–19.4)
Access to electricity (*n* = 456)			
Public network with own meter	269	59.0 (53.4–64.4)	62.3 (53.4–71.2)
Public network with shared meter	161	35.3 (30.2–40.8)	33.0 (24.5–41.5)
Other	26	5.7 (3.6–8.9)	4.7 (1.6–7.7)
Partner status (*n* = 456)			
Partnered	187	41.0 (36.0–46.2)	38.4 (29.6–47.1)
Single	269	59.0 (53.8–64.0)	61.6 (52.9–70.4)
Head of household (*n* = 456)			
Yes	226	49.6 (44.3–54.8)	43.9 (33.8–54.0)
No	230	50.4 (45.2–55.7)	56.1 (46.0–66.2)
Living alone (*n* = 456)			
Yes	28	6.1 (4.1–9.2)	5.9 (0.6–11.2)
No	428	93.9 (90.8–95.9)	94.1 (88.8–99.4)
Number of children living with DW (*n* = 343)			
1 child	127	37.0 (31.0–43.4)	40.7 (29.5–51.9)
2 children	118	34.4 (28.6–40.8)	32.6 (23.3–41.8)
3 or more children	98	28.6 (23.1–34.7)	26.7 (17.8–35.6)
Member of DW’s union (*n* = 456)			
Yes	43	9.4 (6.8–13.0)	7.5 (3.9–11.1)
No	413	90.6 (87.0–93.2)	92.5 (88.9–96.1)
Live-in status (*n* = 456)			
Live-in	16	3.5 (2.0–6.0)	3.4 (0.0–7.2)
Live-out	440	96.5 (94.0–98.0)	96.6 (92.8–100.0)
Number of employers (*n* = 456)			
Single employer	344	75.4 (70.7–79.7)	80.5 (74.2–86.7)
Multiple employers	112	24.6 (20.3–29.3)	19.5 (13.3–25.8)
Employment status (*n* = 456)			
Formal	49	10.7 (7.9–14.4)	9.5 (6.4–12.5)
Informal	407	89.3 (85.6–92.1)	90.5 (87.5–93.6)
Working time arrangement (*n* = 456)			
0 to ≤ 7 h/day	119	26.1 (21.5–31.3)	27.3 (19.1–35.6)
> 7 to ≤ 9 h/day	252	55.3 (49.7–60.7)	54.4 (44.7–64.2)
> 9 h/day	85	18.6 (14.7–23.4)	18.2 (11.7–24.8)
Work experience (*n* = 441)			
0–1 year	11	2.5 (1.2–5.0)	3.5 (0.0–7.8)
1–5 years	173	39.2 (33.8–44.9)	48.2 (37.8–58.7)
> 5 years	257	58.3 (52.6–63.8)	48.3 (37.6–58.9)

Most participants indicated they access potable water and electricity through their own meters; however, 13.4% obtain potable water from alternative sources, such as community pumps with piped lines, water tanks, wells, tanker trucks, or neighbouring households. Additionally, 4.7% access electricity through other connections, mainly through neighbours who share their connection with them through internal agreements. Other participant characteristics are summarized in Table 1.

### Working conditions

Most participants were live-out DWs; only 3.4% live in their employers’ households. Additionally, 80.5% work for a single employer, 90.5% have informal employment status, and 54.4% work more between 7 and 9 h/day (Table 1). Most perform 3–4 tasks as part of their job, and the most common tasks are cleaning, cooking, and laundry (Table 2).

**Table 2:** Counts, percentages and 95% confidence intervals characterizing the domestic work tasks of study participants

Tasks	RDS-II adjusted estimate	
	*n* = 456	% (95 CI)
Cleaning	412	89.9 (85.5–94.3)
Cooking	325	68.5 (59.3–77.8)
Laundry	265	56.7 (46.9–66.5)
Childcare	184	37.0 (28.2–45.8)
Shopping	141	26.9 (20.8–33.1)
Animal care	65	13.0 (8.1–18.0)
Elderly care	53	12.8 (7.4–18.2)
Gardening	34	7.1 (4.7–9.6)
Care for disabled people	9	2.0 (0.0–4.2)
Count of tasks (total)		
Only 1 task	52	13.1 (4.7–21.5)
2 tasks	85	19.2 (13.3–25.0)
3 tasks	126	29.2 (18.5–40.0)
4 tasks	112	22.9 (16.0–29.9)
More than 5 tasks	81	15.6 (10.4–20.7)

We did not observe many statistically significant differences in socio-demographic characteristics or working conditions across DWs who were formally vs. informally employed, except for payment frequency (Table 3). We found that a greater percentage of formal employees receive monthly payments (71.5%, 95% CI: 57.2–85.8) while informal employees are more likely to receive daily, weekly or fortnightly payments (*p* = 0.016) (Table 3).

**Table 3:** Counts, percentages and 95% confidence intervals describing sociodemographic and work-related characteristics of the participants, overall and stratified by employment status

Characteristic	Overall (*n* = 456)			Employment status						
				Formal employment (*n* = 49)			Informal employment (*n* = 407)			*P*-value
	*n*	RDS-II adjusted estimate		*n*	RDS-II adjusted estimate		*n*	RDS-II adjusted estimate		
		**%**	**(95 CI)**		**%**	**(95 CI)**		**%**	**(95 CI)**	
Sociodemographic										
Education										
Less than secondary	132	34.6	(22.5–46.6)	11	25.6	(0.0–51.4)	121	35.4	(22.6–48.3)	0.342
Secondary or higher	232	65.4	(53.4–77.5)	25	74.4	(48.6–100.0)	207	64.6	(51.7–77.4)	
Self-identified ethnicity										
Mestizo	233	54.8	(45.0–64.7)	20	43.4	(20.9–66.0)	213	56.0	(45.7–66.4)	0.397
Indigenous	86	15.1	(5.3–24.9)	9	14.4	(2.1–26.7)	77	15.2	(4.9–25.5)	
Afro Peruvian or Afro descendant	43	7.7	(4.7–10.7)	8	12.0	(0.5–23.4)	35	7.3	(4.2–10.3)	
Other	94	22.3	(14.8–29.9)	12	30.3	(7.2–53.3)	82	21.5	(13.7–29.3)	
Partner status										
Partnered	187	38.4	(29.6–47.1)	18	32.8	(10.7–55.0)	169	39.0	(29.8–48.1)	0.475
Single	269	61.6	(52.9–70.4)	31	67.2	(45.0–89.3)	238	61.0	(51.9–70.2)	
Head of household										
Yes	226	43.9	(33.8–54.0)	21	37.2	(15.6–58.9)	205	44.6	(34.0–55.2)	0.411
No	230	56.1	(46.0–66.2)	28	62.8	(41.1–84.4)	202	55.4	(44.8–66.0)	
Number of children living with DW										
1 child	127	40.7	(29.5–51.9)	13	46.2	(11.0–81.3)	114	40.2	(28.7–51.7)	0.641
2 children	118	32.6	(23.3–41.8)	12	36.7	(7.9–65.5)	106	32.2	(22.7–41.8)	
3 or more children	98	26.7	(17.8–35.6)	8	17.1	(3.8–30.4)	90	27.6	(18.2–37.0)	
Work-related										
Number of employers										
Single employer	344	80.5	(74.2–86.7)	40	86.0	(73.6–98.4)	304	79.9	(73.3–86.5)	0.359
Multiple employers	112	19.5	(13.3–25.8)	9	14.0	(1.6–26.4)	103	20.1	(13.5–26.7)	
Working time arrangement										
0 to ≤ 7 h/day	119	27.3	(19.1–35.6)	8	20.7	(3.7–37.7)	111	28.0	(19.4–36.7)	0.656
> 7 to ≤ 9 h/day	252	54.4	(44.7–64.2)	29	56.6	(33.6–79.6)	223	54.2	(43.9–64.4)	
> 9 h/day	85	18.2	(11.7–24.8)	12	22.7	(0.0–45.9)	73	17.8	(11.0–24.5)	
Payment frequency										
Daily	63	12.5	(7.1–18.0)	3	7.0	(0.0–14.9)	60	13.1	(7.3–18.9)	0.016
Weekly	111	23.4	(15.7–31.0)	7	10.6	(3.1–18.2)	104	24.7	(16.6–32.8)	
Fortnightly	125	28.4	(18.1–38.8)	11	10.8	(3.0–18.6)	114	30.3	(19.4–41.1)	
Monthly	157	35.7	(26.5–44.8)	28	71.5	(57.2–85.8)	129	31.9	(22.5–41.3)	

### Access to labour rights

The labour rights with the highest coverage were timely wage payment (90.0%) and at least one day off per week (94.7%), whereas coverage was lowest for severance pay (3.3%) and provision of payslips (2.8%). When stratified by employment status, coverage of all labour rights was higher among DWs in formal employment. However, fewer than 25% of formal DWs reported coverage for double pay for working on holidays, granting medical leave when needed, severance pay, and payslips (Table 4).

**Table 4:** Counts, percentages and 95% confidence intervals describing access to labour policies, overall and stratified by employment status

Characteristic	Overall (*n* = 456)			Employment status						
				Formal employment (*n* = 49)			Informal employment (*n* = 407)			*P*-value
	*n*	RDS-II adjusted estimate		*n*	RDS-II adjusted estimate		*n*	RDS-II adjusted estimate		
		**%**	**(95 CI)**		**%**	**(95 CI)**		**%**	**(95 CI)**	
Labour policies										
Timely payment										
No	56	10.0	(5.7–14.2)	2	1.7	(0.4–2.9)	54	10.8	(6.3–15.4)	0.026
Yes	398	90.0	(85.8–94.3)	47	98.3	(97.1–99.6)	351	89.2	(84.6–93.7)	
At least one day off per week										
No	24	5.3	(2.4–8.1)	2	8.0	(0.0–29.2)	22	5.0	(2.3–7.6)	0.713
Yes	431	94.7	(91.9–97.6)	47	92.0	(70.8–100.0)	384	95.0	(92.4–97.7)	
Overtime payment										
No	324	71.3	(62.7–80.0)	26	58.8	(38.4–79.2)	298	72.6	(63.6–81.7)	0.122
Yes	132	28.7	(20.0–37.3)	23	41.2	(20.8–61.6)	109	27.4	(18.3–36.4)	
Payment for working on holidays										
Don’t work on holidays	250	53.9	(44.0–63.7)	29	56.4	(31.4–81.4)	221	53.6	(43.2–63.9)	0.501
Regular pay	153	34.6	(25.5–43.7)	9	25.6	(0.0–55.5)	144	35.6	(26.0–45.1)	
Doble pay	53	11.5	(5.3–17.8)	11	17.9	(6.7–29.2)	42	10.8	(4.3–17.4)	
Granting of medical leave										
Never requested	249	57.7	(48.3–67.2)	32	69.1	(46.3–92.0)	217	56.6	(46.5–66.6)	0.34
Not provided	33	7.3	(2.9–11.6)	4	7.0	(0.0–29.6)	29	7.3	(2.9–11.7)	
Provided	174	35.0	(26.3–43.7)	13	23.9	(7.7–40.1)	161	36.1	(26.8–45.4)	
Social security affiliation										
Integral Health Insurance	331	71.5	(60.8–82.3)	29	57.7	(35.4–79.9)	302	73.0	(61.6–84.4)	0.125
Social security	78	16.1	(9.0–23.1)	14	28.2	(6.5–49.8)	64	14.8	(7.6–22.0)	
None	47	12.4	(1.9–22.9)	6	14.2	(1.4–27.0)	41	12.2	(1.1–23.4)	
Employer-paid social security										
No	53	69.6	(46.8–92.5)	4	35.2	(0.0–81.0)	49	76.6	(51.8–100.0)	0.007
Yes	23	30.4	(7.5–53.2)	10	64.8	(19.0–100.0)	13	23.4	(0.0–48.2)	
Retirement pension										
No	413	91.7	(86.3–97.2)	37	79.1	(62.6–95.6)	376	93.0	(87.4–98.7)	0.017
Yes	42	8.3	(2.8–13.7)	12	20.9	(4.4–37.4)	30	7.0	(1.3–12.6)	
Severance pay										
No	436	96.7	(95.4–98.0)	38	77.9	(62.4–93.4)	398	98.7	(97.8–99.5)	0.158
Yes	19	3.3	(2.0–4.6)	11	22.1	(6.6–37.6)	8	1.3	(0.5–2.2)	
Payslips										
No	438	97.2	(91.9–100.0)	42	88.8	(73.6–100.0)	396	98.1	(92.6–100.0)	0.210
Yes	14	2.8	(0.0–8.1)	7	11.2	(0.0–26.4)	7	1.9	(0.0–7.4)	
Paid vacations										
No	336	74.3	(67.4–81.3)	29	58.1	(36.2–80.0)	307	76.0	(69.0–83.1)	0.025
Yes	120	25.7	(18.7–32.6)	20	41.9	(20.0–63.8)	100	24.0	(16.9–31.0)	
Knowledge of Law 31047										
No	388	89.1	(85.4–92.9)	37	79.1	(63.1–95.0)	351	90.2	(86.4–94.0)	0.061
Yes	68	10.9	(7.1–14.6)	12	20.9	(5.0–36.9)	56	9.8	(6.0–13.6)	
Wage										
Weekly payment										
At least 256 PEN/week	41	33.6	(16.9–50.3)	5	64.3	(45.4–83.3)	36	32.2	(15.4–48.9)	0.129
Less than 256 PEN/week	67	66.4	(49.7–83.1)	2	35.7	(16.7–54.6)	65	67.8	(51.1–84.6)	
Fortnightly payment										
At least 513 PEN/fortnightly	60	43.5	(23.7–63.3)	7	57.0	(16.9–97.1)	53	43.0	(21.2–64.8)	0.419
Less than 513 PEN/fortnightly	63	56.5	(36.7–76.3)	4	43.0	(2.9–83.1)	59	57.0	(35.2–78.8)	
Monthly payment										
At least 1025 PEN/day	79	43.9	(29.1–58.7)	21	72.2	(47.7–96.7)	58	37.1	(22.2–52.0)	0.004
Less than 1025 PEN/day	76	56.1	(41.3–70.9)	7	27.8	(3.3–52.3)	69	62.9	(48.0–77.8)	

Lack of timely payment was more prevalent among informally employed DWs (10.8%, 95% CI: 6.3–15.4) compared with those formally employed (1.7%, 95% CI: 0.4–2.9) (*p* = 0.026).

Similarly, DWs in informal employment were less likely to benefit from employer-paid social security (23.4%, 95% CI: 0.0-48.2 vs. 67.8%, 95% CI:19.0-100.0), retirement pension contributions (7.0%, 95% CI: 1.3–12.6 vs. 20.9%, 95% CI:4.4–37.4), and paid vacations (24.0%, 95% CI: 16.9–31.0 vs. 41.9%, 95% CI: 20.0-63.8) than DWs in formal employment (*p* < 0.05). Earning wages below the legal minimum was also more common among informally employed workers across all payment frequencies and was statistically significant among those paid monthly (*p* = 0.004) (Table 4).

### Factors influencing access to labour rights

The most common reasons for not having a written legal contract were that employers did not offer one or explicitly refused to provide it (Table 5). Other open-ended responses included lack of knowledge about contractual requirements, being referred by an acquaintance, trust in the employer, and part-time or hourly work arrangements. For retirement pensions, the main barrier was the perceived unaffordability of mandatory deductions; Other open-ended responses included limited knowledge, lack of awareness of enrolment procedures, absence of a formal contract or payslips, and being an international migrant or a minor. Regarding social security, preference for alternative health insurance schemes was the most frequently cited reason for non-enrolment. Other open-ended responses include barriers included lack of a contract or payslips, employment is not through an company, misconceptions about eligibility, concerns about losing access to the public insurance system, geographic barriers, and perceived poor quality of care or long waiting times. Full responses to the open-ended “Other” question are provided in the Supplementary material 3.

**Table 5:** RDS-II percentage estimates and 95% confidence intervals describing reasons for accessing versus not accessing labour protections

	Reasons for accessing protection	% (95% CI)	Reasons for not accessing protection	% (95% CI)
Written Legal contract	*n* = 47		*n* = 338	
	Employers offer it	30.2 (CI: 8.5–51.8)	Employer didn’t offer/want it	60.1 (CI: 49.2–71.0)
	To ensure job stability	24.8 (CI: 5.1–44.5)	It gives more flexibility	12.6 (CI: 5.3–19.9)
	Influence by family or friends	8.9 (CI: 0.0–17.8)	There is no perceived benefit of it	7.7 (CI: 0.0–15.7)
	Not sure why	7.9 (CI: 2.0–13.8)	To receive a higher salary	1.3 (CI: 0.0–2.9)
	It is required by law	5.9 (CI: 0.0–17.9)	Influenced by family or friends	0.6 (CI: 0.4–0.8)
	Other	22.4 (CI: 0.0–17.9)	Other	17.7 (CI: 13.4–21.9)
Retirement pension	*n* = 42		*n* = 413	
	To have retirement savings	34.9 (CI: 10.0–59.7)	A deduction is unaffordable	17.7 (CI: 7.5–27.8)
	It is required by law	22.7 (CI: 0.0–67.2)	Employer didn’t offer/want it	16.5 (CI: 9.8–23.2)
	Employers offer it	17.8 (CI: 6.8–28.8)	Part-time or multiple employers	8.7 (CI: 4.0–13.5)
	Influenced by family or friends	7.7 (CI: 0.0–17.8)	Prefer alternative retirement savings	7.5 (CI: 3.3–11.7)
	Not sure why	0.7 (CI: 0.0–1.7)	Influenced by family or friends	1.0 (CI: 0.3–1.8)
	Other	16.2 (CI: 3.8–28.7)	Other	48.6 (CI: 38.0–59.1)
Employer-paid social security	*n* = 77		*n* = 377	
	Influenced by family or friends	51.8 (CI: 30.9–72.8)	Prefer a different health insurance	39.2 (CI: 28.6–49.8)
	To protect personal and family health	16.0 (CI: 0.0–32.8)	Employer didn’t offer/want it	16.2 (CI: 9.5–22.8)
	It is required by law	13.3 (CI: 0.0 -35.8)	Part-time or multiple employers	7.9 (CI: 3.1–12.8)
	Employers offer it	8.1 (CI: 0.3–16.0)	Prefers cash contribution rather than insurance	4.0 (CI:1.6–6.4)
	Not sure why	3.1 (CI: 0.0–8.6)	Considers she doesn’t need a health insurance	3.0 (CI: 0.0 -7.9)
	Other	7.6 (CI: 2.1–13.2)	Other	29.8 (CI: 17.9–41.6)

## Discussion

### Key findings

Paid DWs in our study are predominantly women aged 19–45 years, self-identifying as mestizo, non-migrants, single, and frequently heads of household. Most work as live-out DWs, typically for a single employer, under informal employment and working between 7 and 9 h/day. When stratified by employment status, informally employed DWs were less likely to receive monthly and timely payments, earn the minimum legal monthly wage, and benefit from employer-paid insurance, retirement pension, and paid-vacations. Despite the enactment of a new domestic workers’ law in 2021, full coverage of labour rights remains limited. The most frequently reported reasons for not accessing labour rights include employers’ failure to offer them, limited perceived benefits by DWs, preference for other insurance systems, part-time work or having multiple employers, and the absence of formal contracts or payslips.

Peru’s population is highly diverse, with 60% identifying as mestizo, 26% as Indigenous, and 3.6% as Afro-Peruvian or Afro-descendant [26]. In our sample, the share of Afro-descendant workers was higher than the national proportion, suggesting a possible over-representation of this group in domestic work. A previous analysis found that, among Peruvian female workers identified as Afro-descendant, 6% were employed in domestic services, compared with 5% in non-domestic personal services, such as beauty services, and 3% in non-personal services, such as financial services [27]. This overrepresentation may be partly explained by the characteristics of domestic work; however, no definitive conclusions can be drawn. Nevertheless, this pattern needs further investigation, particularly considering the colonial legacies that have historically shaped this occupational group.

Since many women DWs sustain their households, labour rights gaps have consequences beyond the individual worker. It may affect family wellbeing and increase intergenerational vulnerability. Many women may accept precarious working conditions because they are responsible for sustaining their households, including children and other dependents with daily care needs. In our study, 343 of 456 participants had children. Although differences by employment status were not statistically significant, women in informal employment more often reported having three or more children than those in formal employment (27.6% vs. 17.1%). This pattern should be interpreted cautiously, but it suggests that caregiving responsibilities may constrain employment options and bargaining power. Paid domestic work may represent one of the few viable employment options available to them, particularly when labour market opportunities are constrained by gendered care responsibilities, low income, and limited access to formal employment [28].

Our findings are consistent with prior literature. Globally, it is estimated that 49.9% of DW’s are legally covered by social security; however, in reality, one out of five DWs benefits from it [1]. The sector’s high informality and instability exclude workers from social protection, leading to precarious conditions for them and their families [5, 29]. The varying combinations of access or lack thereof to formality, job stability, and social protection contribute to the characterization of the domestic labour market as unstructured [5, 30]. Factors that contribute to this situation include the private domain of the workplace and the employer’s discretion to hire, fire and define the tasks [30]. The employer is simultaneously the regulator and recipient of services. Labour regulatory agencies should consider that domestic work is a private-household-based sector where traditional labour inspections are not feasible mechanisms. Instead, they should consider alternatives such as complaint-based systems, anonymous reporting, or simplified compliance tools.

We found that most Peruvian DWs remained informally employed despite the existence of Law 31,047, suggesting persistent gaps between legal reform and implementation. This finding highlights the need to distinguish between the legal recognition of labour rights and workers’ effective access to those rights. In practice, written contracts and payslips are key documents for making labour protections enforceable. Without written evidence of the employment relationship, DWs may face difficulties claiming minimum wages, social security, pension contributions, paid holidays, or other benefits. Thus, informality is not merely a contractual category; it is associated with systematic exclusion from multiple forms of labour and social protection. Therefore, closing the implementation gap requires more than legal recognition. It requires practical mechanisms that make employment relationships visible, documentable, and enforceable.

Relying solely on employers’ goodwill is insufficient. Ensuring that DWs actively understand and defend their labour rights is critical. We found that the main reason for not accessing employer-paid social security was because DWs preferred a different health insurance, the Integral Health Insurance (Seguro Integral de Salud or SIS, in Spanish). Some open-ended responses stated they perceived healthcare in social security is poor and have long waiting times. Yet it overlooks the fact that social security affiliation extends beyond access to healthcare. Legally, formal employees affiliated with the social security system benefit from essential protections, including paid maternity leave, coverage for work-related accidents, disability benefits, and funeral support [31]. Lack of knowledge about these entitlements perpetuates exclusion. An analysis of data from social security affiliates shows that insured individuals use other institution services because they are unaware of the services provided or the rights to which they are entitled [32]. Therefore, it is essential to raise awareness about these broader protections and to empower DWs to claim their rightful inclusion in social security systems, as is the case for any other salaried worker. At the same time, formalization policies should consider how DWs perceive and experience the value of labour benefits. They could clarify the concrete advantages of formal employment and avoid penalizing workers who rely on alternative coverage.

Beyond limited knowledge and awareness of labour rights, access to retirement pensions among DWs is constrained by a structural trap of informality and precarious employment.

We found that the main barriers to accessing retirement pensions include a lack of formal employment relationships, the absence of payslips, and the unaffordability of pension contributions. These factors operate in a vicious cycle: informal employment precludes access to payslips and pension schemes, while the absence of pension coverage further entrenches workers in the informal labour market. In our study, some DWs reported that saving for old age was not feasible because immediate income needs take precedence, particularly given wages below the legal minimum. This dynamic contributes to the intergenerational and life-course perpetuation of poverty through low earnings in the present and lack of income security in older age [12]. These vulnerabilities were exacerbated during the COVID-19 pandemic, when many domestic workers were dismissed and unable to return to employment, highlighting the long-term consequences of exclusion from social protection systems [1].

The government also plays a central role in extending DW’s rights and compliance with the law. Some entitlements for DWs with part-time job or multiple employers are particularly unclear for the heterogeneous working conditions and varied employment relationship [9]. This gap could be addressed by legal reform. These reforms will likely need to be driven by DWs themselves and their unions, as has historically been the case [33, 34]. Government responsibility also lies in creating a balanced framework that deters noncompliance through sanctions while also facilitating compliance through accessible support measures for employers. To address implementation gaps, Peru can look to successful regional models. Countries like Argentina, Brazil, and Mexico have introduced simplified mechanisms, such as wage and social security calculators [35]. Costa Rica and Ecuador have promoted formalization by allowing tax deductions for employer contributions [35]. Uruguay offers perhaps the most comprehensive model, combining simplified enrolment processes with tripartite awareness campaigns and strengthened inspection systems [36].

### Strengths and limitations

This study contributes to an existing literature base by providing context-specific evidence on the characteristics of domestic work in Peru, using a survey explicitly designed for this purpose. Our analysis draws on one of the largest samples of DWs to date, particularly given that this population is difficult to reach because of the nature of their working conditions.

Another strength of this study is its participatory approach: DWs and their organizations were actively involved in survey design and in nominating the initial seeds for recruitment.

Our study also had several limitations. Findings may be affected by self-report bias, including recall bias or social desirability bias [37]. However, this limitation does not invalidate the study, as self-reported data remain the most appropriate and widely accepted approach for capturing employment conditions, labour rights coverage, and lived experiences in highly informal and underregulated sectors [38]. For questions on reasons for accessing or not accessing labour rights, we recognise that predefined categories may capture situations with different implications; for example, not knowing about a legal right differs from an employer explicitly not offering it. These issues were further explored through interviews with DWs and employers, which will be reported in a separate publication.

Although representativeness is limited by the sampling design and the focus on only three study sites, recruitment was diversified through RDS, with DWs themselves facilitating the process. However, due to the design effects of RDS and the inflated variances, comparisons between working conditions and labour rights do not reach the traditional levels of statistical significance. Additionally, this study was not originally powered to detect differences between formal and informal statuses. Nonetheless, we note that the magnitude of these differences is often substantial, which is particularly relevant given the limited information available for DWs. Despite the use of a RDS design, we do recognize that the sample size of DWs in formal employment is small (*n* = 49), which may affect estimates and CI of the statistical analysis; however, this small number reflects the scarcity of formal employment in this sector.

Analyses were descriptive and relied on bivariate comparisons. Consequently, the observed associations should not be interpreted as independent effects, as they may be influenced by unmeasured confounding. We intentionally adopted this approach because the study was designed to characterize labour rights and social protection among DWs and generate hypotheses for future research. Given the large number of outcomes examined and ongoing methodological challenges related to regression modelling with RDS data, we did not undertake multivariable analyses. Future studies should build on these findings by evaluating selected outcomes using multivariable methods appropriate for RDS-derived samples.

## Conclusions

Despite recent legal reforms, DWs in Peru, particularly those in informal employment, remain largely excluded from effective labour rights coverage. Our findings underscore the need for practical mechanisms to guarantee fair working conditions for women DWs. This includes simplifying documentation, making formalization and benefits procedures more accessible, and developing enforcement mechanisms appropriate for employment relationships in private households. Future research should examine how gender, education, migration, motherhood, and household responsibilities intersect to shape DWs’ employment conditions and access to rights. Expanding labour rights coverage in this sector is essential not only to improve working conditions, but also to reduce socioeconomic vulnerability among a historically undervalued workforce. 

## Supplementary Information

Supplementary Material 1.

Supplementary Material 2.

Supplementary Material 3.

## Data Availability

Given the nature of the data and REB approval, the data supporting the findings of this study are available from the corresponding author, JTM, upon reasonable request.
