# Supplementary material for: Working conditions and labour rights coverage of women domestic workers in Peru: a respondent-driven sampling study
Source: BMC Public Health. 2026 Jul 14;26:2877. doi: 10.1186/s12889-026-28527-2 (PMC13647820; doi:10.1186/s12889-026-28527-2)
Supplement: Supplementary file 1 [file 12889_2026_28527_MOESM1_ESM.pdf]

ANITA: "Abordando los Desafíos y Limitaciones de las Políticas de Protección Social para las Trabajadoras del Hogar en el Perú"

Módulo 1 - Características Sociodemográficas y Económicas

SECCIÓN 1 - EDUCACIÓN Y ETNICIDAD

Duracion ______________________________

1 ¿Cuál fue el año o grado de estudios más alto que aprobó? (Respuesta única, espontánea)

- ◯ Sin estudios
- ◯ Inicial
- ◯ Primaria incompleta
- ◯ Primaria completa
- ◯ Secundaria incompleta
- ◯ Secundaria completa
- ◯ Superior No Universitaria
- ◯ Superior Universitaria
- ◯ Posgrado
- ◯ No sabe/ No recuerda

2 ¿Actualmente está asistiendo a la escuela, colegio, instituto superior o universidad? (Respuesta única, espontánea)

- ◯ A la primaria
- ◯ A la secundaria
- ◯ Al instituto superior o pedagógico
- ◯ A la universidad
- ◯ No asiste a ninguno

3 (Si NO respondió la opción 5 en la pregunta anterior) ¿En qué año de estudios se encuentra? (Respuesta única, espontánea)

- ◯ Primero
- ◯ Segundo
- ◯ Tercero
- ◯ Cuarto
- ◯ Quinto
- ◯ Sexto

4 (Si responde 2, 3, 4 o 5 en la p.1) ¿Cuál fue la principal razón por la que usted dejó de estudiar? (Respuesta única, espontánea)

- ◯ Quedó embarazada
- ◯ Se casó / Se unió (Unión de hecho)
- ◯ Tenía que cuidar a niño/as más pequeños
- ◯ Tenía que ayudar en el negocio familiar (p.ej. ayudar en la chacra)
- ◯ Falta de dinero / Necesitaba trabajar
- ◯ Enfermedad
- ◯ No quiso estudiar
- ◯ Escuela muy lejos/ No había escuela
- ◯ Otro ______
- ◯ No sabe

5 ¿Cuál fue el año o grado de estudios más alto de su madre? (Respuesta única, espontánea)

- ◯ Sin estudios
- ◯ Inicial
- ◯ Primaria incompleta
- ◯ Primaria completa
- ◯ Secundaria incompleta
- ◯ Secundaria completa
- ◯ Superior No universitaria
- ◯ Superior Universitaria
- ◯ Posgrado
- ◯ No sabe/ No recuerda

6 ¿Cuál fue el año o grado de estudios más alto de su padre? (Respuesta única, espontánea)

- ◯ Sin estudios
- ◯ Inicial
- ◯ Primaria incompleta
- ◯ Primaria completa
- ◯ Secundaria incompleta
- ◯ Secundaria completa
- ◯ Superior No universitaria
- ◯ Superior Universitaria
- ◯ Posgrado
- ◯ No sabe/ No recuerda

Ahora me gustaría que usted lea en voz alta la siguiente frase: (muestre la cartilla 1 a la entrevistada).
Nota: Ver instrucciones para esta pregunta y cartilla de lectura al final de este documento.

"Mi Perú es maravilloso. Tiene ríos, lagos, lagunas, montañas, plantas y muchos animales. Su clima es variado según sus regiones".

SOBRE PREGUNTA 7:

OBJETIVO: Conocer si la informante con nivel de instrucción primaria o menos, sabe leer o no; así como determinar la cobertura de enseñanza por la modalidad de alfabetización (sistema no escolarizado).

DILIGENCIAMIENTO DE LA PREGUNTA 7:

Utilice la cartilla de lectura para comprobar si la entrevistada sabe leer o no, y con qué facilidad lee, cuando sólo accedieron al nivel de educación primaria o menos y de acuerdo al resultado de la prueba seleccione uno de los códigos del "1" al "4" y si la persona es invidente o tiene dificultad que le impida leer (miopía, falta de lentes adecuados, etc.) pese a saber leer seleccione el código "5"

7	Si la entrevistada no puede leer toda la frase, repregunte, ¿Puede Ud. leer parte de alguna la frase?<br><br>Nota: Ver instrucciones para esta pregunta y cartilla de lectura al final de este documento.	◯ No puede leer<br>◯ Puede leer solo parte de la frase<br>◯ Puede leer la frase<br>◯ No hay tarjeta en el idioma requerido ______<br>◯ Ciega/Problemas visuales
8	¿Cuál es el idioma o lengua materna con el que aprendió hablar en su niñez? (Respuesta única, espontánea)	◯ Castellano<br>◯ Quechua<br>◯ Aimara<br>◯ Otra lengua nativa u originaria ______<br>◯ Lengua extranjera ______
9	¿Qué idioma hablan habitualmente en su hogar (donde vive con su familia)? (Respuesta única, espontánea)	◯ Castellano<br>◯ Quechua<br>◯ Aimara<br>◯ Otra lengua nativa u originaria ______<br>◯ Lengua extranjera ______
10	¿Qué idioma hablan habitualmente en su centro de labores principal? (Respuesta única, espontánea)	◯ Castellano<br>◯ Quechua<br>◯ Aimara<br>◯ Otra lengua nativa u originaria ______<br>◯ Lengua extranjera ______
11	Por sus costumbres y sus antepasados, ¿Usted se siente o considera...? (Respuesta única, espontánea)	☐ Quechua<br>☐ Aimara<br>☐ Nativa o indígena de la Amazonía<br>☐ Perteneciente o parte de un pueblo indígena u originario<br>☐ Afroperuana o Afrodescendiente/Negra /Morena/ Zamba/Mulata<br>☐ Blanca<br>☐ Mestiza<br>☐ Asiática descendiente<br>☐ Otro ______<br>☐ No sabe/No responde
12	¿Usted diría que profesa la siguiente religión? (Respuesta única, espontánea)	◯ Catolicismo<br>◯ Cristiana (Evangélica)<br>◯ Cristiana (Protestante, Adventista)<br>◯ No profeso ninguna religión<br>◯ Otra religión ______

SECCIÓN 2 - LUGAR DE RESIDENCIA

13 ¿Su residencia actual es el mismo lugar donde usted trabaja?
◯ Sí
◯ No

14 ¿En qué lugar trabaja como ocupación principal? (Leer categorías)

1. Departamento: ______ 2. Provincia: ______

3. Distrito: ______ 4. Centro Poblado: ______

15 ¿En qué lugar vive con su familia? (Leer categorías)

1. Departamento: ______ 2. Provincia: ______ 3. Distrito: ______ 4. Centro Poblado: ______

16 ¿Cuánto tiempo tiene usted viviendo continuamente en su residencia actual?
☐ Años ______
☐ Meses ______
☐ Siempre

17 Antes de que usted viniera a vivir aquí, ¿su hogar se localizaba en una ciudad, en un pueblo, o en el campo? (Respuesta única)
◯ Ciudad
◯ Pueblo
◯ Campo

18 ¿Nos podría especificar dónde vivía antes que usted viniera a vivir aquí? (Leer categorías)

1. País ______ 2. Departamento: ______ 3. Provincia: ______

4. Distrito: ______ 5. Centro Poblado: ______

19 Entre marzo de 2020 a diciembre de 2021, ¿usted cambió de lugar de residencia?
◯ Sí
◯ No

20 ¿Nos podría especificar a dónde cambió de lugar de residencia en ese período? (Leer categorías)

1. País ______ 2. Departamento: ______ 3. Provincia: ______

4. Distrito: ______ 5. Centro Poblado: ______

21 ¿Usted considera que este cambio de residencia se debió a la pandemia del por la COVID-19 (coronavirus)?
◯ Sí
◯ No

22 ¿Usted volvió a residir en el lugar donde vivía antes de la pandemia por la COVID-19?
◯ Sí
◯ No

SECCIÓN 3 - COMUNICACIONES

23 ¿Con qué frecuencia usted accede a internet (ya sea desde un celular o una computadora)? (Respuesta única, leer alternativas)

- ◯ Casi todos los días
- ◯ Una vez por semana
- ◯ De vez en cuando
- ◯ Nunca

24 ¿Desde qué equipo accede a internet? (Respuesta única, leer alternativas)

- ◯ Desde mi celular
- ◯ Desde el celular de alguien cercano
- ◯ Desde una computadora en casa
- ◯ Desde una computadora en el trabajo
- ◯ Desde un lugar público, cabinas de internet, librería, etc.

25 En los últimos 12 meses, ¿usted ha usado un celular o alguna computadora para lo siguiente? (Leer alternativas, respuesta múltiple)

- ☐ Revisar noticias de familiares y/o conocidos
- ☐ Revisar noticias del país y el mundo
- ☐ Buscar información médica para usted
- ☐ Usar email/correo electrónico para comunicación en general
- ☐ Usar internet (email/videoconferencia) para comunicarse con un profesional de salud o centro de atención en salud
- ☐ Recibir o revisar resultados médicos
- ☐ Hacer citas con algún proveedor de atención en salud

26 Algunas veces las personas usan el internet para conectarse con otras personas a través de aplicaciones de mensajería (WhatsApp, Messenger, Telegram), además de las redes sociales (como Facebook, Twitter, Instagram, etc.). En los últimos 12 meses, ¿usted ha usado un celular o alguna computadora para lo siguiente? (Leer alternativas, respuesta múltiple)

- ☐ Para comunicarse con familiares o amistades por mensajería como WhatsApp, Messenger, etc.
- ☐ Para visitar una red social como Facebook, Twitter, etc.
- ☐ Para compartir información de salud que se difunde en redes sociales (tales como Facebook, Twitter, etc.)
- ☐ Para participar en foros o reuniones virtuales sobre salud
- ☐ Para participar en foros o reuniones virtuales sobre derechos laborales o temas relacionados a trabajadoras del hogar
- ☐ Para mirar videos en YouTube sobre temas relacionados a salud

¿Cuál es la aplicación o red social que usa con mayor frecuencia para comunicarse y/o acceder a información? ¿Y la segunda más frecuente? ¿Y la tercera más frecuente? (Espontánea, indicar número 1, 2 o 3 de acuerdo a preferencia)

	1er lugar	2do lugar	3er lugar
27.1 WhatsApp	○	○	○
27.2 Facebook	○	○	○
27.3 Instagram	○	○	○
27.4 Twitter	○	○	○
27.5 TikTok	○	○	○
27.6 YouTube	○	○	○
27.7 Otro	○	○	○

Especifique la red social que utiliza

____

SECCIÓN 3 - COMUNICACIONES

	¿Qué aplicación o red social usa con mayor frecuencia para comunicarse y/o acceder a información?	◯ WhatsApp<br>◯ Facebook<br>◯ Instagram<br>◯ Twitter<br>◯ TikTok<br>◯ YouTube<br>◯ Otro ______
28	En su tiempo libre (fuera del trabajo) es más probable que usted vaya a un / una: (Leer alternativas, respuesta única) *Encuestador/a: En caso haya más de una alternativa de respuesta, solicitarle a la persona entrevistada que indique aquella actividad que realiza con mayor frecuencia.	◯ Iglesia<br>◯ Actividad artística (música, danza, museos, etc.)<br>◯ Conversatorios o charlas informativas sobre algún tema en particular<br>◯ Centro de estudios<br>◯ Local partidario o sindical<br>◯ Visita a su familia o amistades<br>◯ Paseo sola/familia/amistades<br>◯ Practica de algún deporte<br>◯ Usar Internet (para navegar, comunicarse, ver videos, etc.)<br>◯ Actividad económica o productiva<br>◯ Descanso/ reposo / duerme<br>◯ No tengo tiempo libre<br>◯ Ninguno de los anteriores<br>◯ Otra actividad. Especifique____________
	Por favor, especifique	__________________________________

SECCIÓN 4 - CARACTERÍSTICAS DEL HOGAR

29 Actualmente, ¿usted vive sola?

- ◯ Sí
- ◯ No

30 ¿Me podría indicar con cuántas personas vive normalmente en su hogar (donde vive con su familia), sin contar los que se encuentran de visita?

____

31 Incluida usted, ¿Cuántas personas conforman su hogar?

____

32 Incluida usted, ¿cuántas personas aportan económicamente a su hogar?

____

33 ¿Cuántas personas menores de 14 años habitan en su hogar?

____

34 ¿Me podría indicar quién es el jefe/jefa de hogar en su casa? (Respuesta espontánea, múltiple)

- ☐ Yo misma
- ☐ Esposo/a, compañero/a
- ☐ Hijo/a
- ☐ Papá
- ☐ Mamá
- ☐ Tío/Tía
- ☐ Yerno/Nuera
- ☐ Abuelo/Abuela
- ☐ Suegro/Suegra
- ☐ Empleador
- ☐ Otro ______

35 Ahora, me gustaría preguntarle acerca de todas las hijas e hijos que usted ha tenido durante su vida. ¿Ha tenido alguna hija o hijo nacido vivo? (Si responde afirmativamente, indicar cuántos)

- ◯ No tuvo hijos
- ◯ Sí tuvo hijos nacidos vivos ______
- ◯ Sí tuvo hijos fallecidos ______

36 ¿Me puede indicar sus edades actuales? (separe las edades con coma)

____

37 ¿Tiene alguna hija o hijo que esté viviendo con usted actualmente? (Si responde afirmativamente, indicar cuántos)

- ◯ Sí ______
- ◯ No

38 ¿Su casa es ... ? (Leer opciones, respuesta única)

- ◯ Propia (con titulo de propiedad o constancia de posesión)
- ◯ Alquilada
- ◯ Hipotecada
- ◯ Cedida
- ◯ De familiares
- ◯ Otro ______

39 ¿Quién o quiénes aportan para el pago del alquiler mensual o el pago de la hipoteca? (Respuesta espontánea, múltiple)

- ☐ Sólo yo
- ☐ Esposo/a, compañero/a
- ☐ Hijo/a
- ☐ Yerno/nuera
- ☐ Nieto/Nieta
- ☐ Padre/Madre
- ☐ Suegro/Suegra
- ☐ Hijo adoptado/Hijo de crianza
- ☐ Otro familiar

40 ¿Cuál es el material predominante en los pisos de su vivienda? (Respuesta espontánea, múltiple)

- ◯ Piso natural: Tierra / Arena
- ◯ Piso rústico: Cemento sin pulir o pulido / Tablones sin pulir
- ◯ Losetas / Terrazos, mayólicas, cerámicos
- ◯ Parquet / Alfombra / Mármol / Porcelanato
- ◯ No sabe/No contesta [NO LEER]

41 ¿Cuántas habitaciones tiene en su hogar que actualmente se usan exclusivamente para dormir?

____

42 ¿De dónde proviene el agua que utiliza para cocinar y asearse actualmente? (Leer opciones, respuesta única)

- ◯ Red pública con medidor propio
- ◯ Red pública, con medidor compartido
- ◯ Red pública sin medidor
- ◯ Pozo natural
- ◯ Río, vertiente, lago
- ◯ Camión cisterna
- ◯ Otra Fuente ______

43 ¿Cuál es el tipo de servicio higiénico que tiene en su vivienda? (Leer opciones, respuesta única)

- ◯ Water / inodoro conectado a la red pública de desagüe
- ◯ Letrina / silo / pozo séptico
- ◯ No tiene servicio higiénico
- ◯ No sabe / No responde

44 ¿De dónde proviene la energía eléctrica que utiliza actualmente? (Leer opciones, respuesta única)

- ◯ Red pública con medidor propio
- ◯ Red pública con medidor compartido
- ◯ Red pública sin medidor
- ◯ De un generador propio /comunitario
- ◯ Otra fuente ______
- ◯ No tiene energía eléctrica

Comentarios/observaciones del encuestador (opcional)

____

ANITA: "Abordando los Desafíos y Limitaciones de las Políticas de Protección Social para las Trabajadoras del Hogar en el Perú"

Módulo 2 - Condiciones laborales

DURACION

____

Hora de inicio de la segunda ronda

____

---

1 ¿La remuneración que usted percibe como trabajadora del hogar constituye su principal fuente de ingresos? (Leer opciones, respuesta única)

- ◯ Sí es mi principal fuente de ingresos
- ◯ No es mi principal fuente de ingresos
- ◯ No percibo ingresos económicos como trabajadora del hogar

---

TERMINAR ENCUESTA Agradecer a la participante y dar por terminada la entrevista

SECCIÓN 1 - HORARIOS DE TRABAJO

2 ¿Desde hace cuánto tiempo labora en su trabajo principal actual como trabajadora del hogar, es decir, donde más horas trabaja?

Años: ______ y Meses: ______ y Días: ______

3 En promedio, ¿cuántas horas al día labora como trabajadora del hogar? (restando el tiempo que usa para almorzar o tomar refrigerios) Indicar número de horas y minutos (Si el encuestado tiene dificultades en calcular pedirle horas promedio del día, y ayudar a calcular)

______ ______
Escribir en texto: ______
(Registrar minutos solo cuando sea necesario)

4 En una semana típica de trabajo, ¿cuántos días trabaja a la semana? (Respuesta única)

◯ 1 ◯ 2 ◯ 3 ◯ 4
◯ 5 ◯ 6 ◯ 7

5 En una semana típica de trabajo, ¿Qué días de la semana trabaja usted? (Espontánea y múltiple)

☐ Lunes
☐ Martes
☐ Miércoles
☐ Jueves
☐ Viernes
☐ Sábado
☐ Domingo
☐ Son días irregulares o no fijos o movibles

6 Según su contrato o acuerdo laboral en su centro de trabajo principal (Recoger la información de la vivienda donde laboren más horas a la semana):

¿Cuántas horas debe trabajar al día? ¿Y a la semana?
______ ______

7 ¿Trabaja usted los días feriados? (Leer opciones, respuesta única)

◯ No trabajo en días feriados
◯ Trabajo los feriados y percibo el mismo pago que cualquier día normal
◯ Trabajo los feriados y percibo un doble pago por ese día

8 Durante la semana, ¿tiene usted por lo menos 24 horas de descanso continuas, es decir un día completo?

◯ Sí
◯ No

9 Durante su jornada de trabajo, ¿qué comidas diarias toma en su centro de labores? (Leer opciones, respuesta múltiple)

☐ Desayuno
☐ Almuerzo
☐ Lonche
☐ Cena
☐ Ninguna

10 ¿Qué comidas le proporciona su empleador? (Leer opciones, respuesta múltiple)

☐ Desayuno
☐ Almuerzo
☐ Lonche
☐ Cena
☐ Ninguna

11 ¿Es la misma comida que comen los miembros de la familia donde usted trabaja o no?

◯ Generalmente es la misma comida
◯ Generalmente es una comida diferente

¿Usted trabajó el 30 de marzo (Día Trabajadoras y Trabajadores del Hogar) del presente año?

◯ Sí
◯ No

12 En promedio, ¿cuánto tiempo destina cada día para tomar sus alimentos durante su horario de trabajo? (Especificar cantidad de horas y/o minutos diarios por cada comida, completar con ceros si es necesario)

Desayuno: ______ ______
Almuerzo: ______ ______
Lonche: ______ ______
Cena: ______ ______

13 En el último mes, ¿alguna vez tuvo menos de 12 horas libres entre el final de un día de trabajo y el inicio de la siguiente jornada de trabajo?

◯ Sí
◯ No
◯ No sabe/ No contesta

SECCIÓN 2 - CONDICIONES DEL LUGAR DE TRABAJO

14	Su contrato o acuerdo de trabajo como trabajadora del hogar es del tipo: (Leer opciones, respuesta única)	◯ Con residencia (cama adentro)<br>◯ Sin residencia (cama afuera)
15	¿En qué lugar pernocta/duerme en su principal centro de labores? (Leer opciones, respuesta múltiple)	◯ En una habitación independiente<br>◯ En el sillón de la sala<br>◯ En otro lugar adaptado de la vivienda (biblioteca, estudio, habitación de lavado, almacén, etc.)<br>◯ Comparte habitación con algún miembro de la familia<br>◯ Comparte habitación con otra trabajadora del hogar<br>◯ Otro ______
16	¿Tiene un baño exclusivo para usted en su centro de trabajo?	◯ Sí<br>◯ No

¿Qué tan satisfecha se siente con los siguientes aspectos del lugar donde usted pernocta/duerme ?

	Muy insatisfecha	Insatisfecha	Ni satisfecha ni insatisfecha	Satisfecha	Muy satisfecha
17.1 Habitabilidad	○	○	○	○	○
17.2 Confortabilidad/comodidad	○	○	○	○	○
17.3 Privacidad	○	○	○	○	○
17.4 Tamaño	○	○	○	○	○
17.5 Ventilación	○	○	○	○	○
17.6 Iluminación	○	○	○	○	○
17.7 Servicios higiénicos	○	○	○	○	○

SECCIÓN 2 - CONDICIONES DEL LUGAR DE TRABAJO

18 Como trabajadora del hogar, ¿trabaja en una o más casas/viviendas?

◯ Solamente en una casa
◯ En más de una casa ______

SECCIÓN 3 - ASEGURAMIENTO PREVISIONAL Y DE SALUD

19	¿A qué sistema de jubilación está afiliada? (Leer opciones, respuesta única)	◯ Oficina de Normalización Previsional (ONP)<br>◯ Administradora de fondos de pensiones (AFP)<br>◯ Otro<br>◯ No está afiliada<br>◯ No sabe/No responde
20	Actualmente, ¿Le descuentan o usted aporta a algún sistema de jubilación (ONP/AFP/Otros)?	◯ Sí<br>◯ No
21	¿A qué sistema de salud está afiliada? (Leer opciones, respuesta única)	◯ Seguro Integral de Salud (SIS)<br>◯ ESSALUD<br>◯ Seguro de la policía o FFAA.<br>◯ Seguro privado / Entidades prestadoras de salud (EPS)<br>◯ Otro ______<br>◯ No está afiliada<br>◯ No sabe/No responde
22	Actualmente, ¿Te descuentan o usted aporta a algún sistema de salud?	◯ Sí<br>◯ No
23	Si es´ta afialiada a ESSALUD, ¿Es su empleador quien le paga el seguro para que acceda a este servicio?	◯ Sí<br>◯ No
24	Al margen de si está afiliada o no, cuando usted necesita atención médica, ¿a dónde acude generalmente para realizar su consulta de salud? (Múltiple, espontánea)	☐ Puesto / centro de salud MINSA<br>☐ Posta / policlínico ESSALUD<br>☐ Hospital MINSA<br>☐ Hospital del Seguro (ESSALUD)<br>☐ Hospital de las FF.AA. y/o Policía Nacional<br>☐ Hospital de la Solidaridad (SISOL)<br>☐ Consultorio médico particular<br>☐ Clínica particular<br>☐ Farmacia o botica<br>☐ En su domicilio (del paciente)<br>☐ Otro ______<br>☐ No buscó atención
25	¿Por qué no acudió a los establecimientos de salud?	☐ No tuvo dinero<br>☐ Se encuentra lejos<br>☐ Demoran mucho en atender<br>☐ No confía en los médicos<br>☐ No era grave/ no fue necesario<br>☐ Prefiere curarse con remedios caseros<br>☐ No tiene seguro<br>☐ Se auto recetó o repitió receta anterior<br>☐ Falta de tiempo<br>☐ Por el maltrato del personal de salud<br>☐ El/La empleador/a no dio permiso<br>☐ Otro ______

SECCIÓN 4 - CUMPLIMIENTO DE DERECHOS LABORALES Y BENEFICIOS SOCIALES

26 ¿Qué tipo de formalidad laboral tiene usted con su empleador? (Leer opciones, respuesta única)

◯ Tiene un contrato de trabajo registrado en el Ministerio de Trabajo y Promoción del Empleo
◯ Tiene un contrato de trabajo legalizado notarialmente
◯ Tiene un contrato de trabajo simple por escrito
◯ Tiene solamente un acuerdo verbal
◯ Tiene un contrato con un service o agencia de empleo que subcontrata/terceriza mis servicios
◯ No tiene ningún tipo de contrato o acuerdo

---

27 Sobre la remuneración por su trabajo como trabajadora del hogar, ¿cuál es la modalidad de pago?

◯ Recibe pago en efectivo
◯ Recibe transferencia bancaria
◯ Ambas modalidades
◯ Otra modalidad ______

---

28 Sobre la remuneración por su trabajo como trabajadora del hogar, ¿con qué frecuencia recibe su remuneración? (Leer opciones, respuesta única)

◯ Al día
◯ A la semana
◯ Cada quincena
◯ Mensualmente
◯ De manera irregular

---

29 Pensando en su remuneración MENSUAL por su trabajo como trabajadora del hogar. ¿Cuánto es lo que percibe en promedio al MES? (Respuesta única, espontánea)

____

---

30 ¿Recibió gratificaciones de acuerdo a ley por su trabajo, durante el año 2022? (Leer opciones, respuesta única)

◯ No, ninguna gratificación
◯ Sí, una gratificación al año
◯ Sí, dos gratificaciones al año (julio y diciembre)
◯ No trabajó el año 2022 como trabajadora del hogar

---

31 Respecto a sus vacaciones, usted diría que: (Leer alternativas, respuesta única)

◯ Ya ha tomado vacaciones el último año de servicios
◯ Ya cumplió un año de servicios y ha acordado con su empleador sus vacaciones para el presente año
◯ Este año cumple un año de servicios
◯ No ha tomado vacaciones el último año por desconocimiento de la ley
◯ No ha tomado vacaciones el último año por negativa de su empleador
◯ El empleador le otorgó o le otorgará vacaciones, pero no remuneradas

---

32 Si tuvo un trabajo como trabajadora del hogar anterior al actual, ¿Recibió CTS al terminar su contrato o acuerdo de trabajo?

◯ Sí
◯ No
◯ No sabe
◯ No tuvo un trabajo anterior como trabajadora del hogar

---

33 ¿Sabía usted que la CTS se deposita en los meses de mayo y noviembre?

◯ Sí
◯ No

---

34 ¿Sabe usted a cuánto asciende la CTS? (Espontánea, respuesta única)

◯ Un sueldo completo / Medio sueldo en cada depósito
◯ Otra respuesta
◯ No sabe/No responde

---

35 ¿Alguna vez ha necesitado un descanso médico? (Leer alternativas, respuesta única)

◯ Sí, y fue facilitado por el empleador
◯ Sí, pero no fue facilitado por el empleador
◯ Nunca necesitó un descanso médico

36 ¿Alguna vez ha necesitado atención médica durante la jornada laboral? (Leer alternativas, respuesta única)

- ◯ Sí, y pudo acudir oportunamente al médico
- ◯ Sí, pero debió esperar unos días hasta que el empleador le diera permiso para acudir al médico
- ◯ Sí, pero debió esperar al día de descanso para acudir al médico
- ◯ Sí, pero nunca acudió al médico
- ◯ Nunca necesitó atención médica

37 En los últimos cinco años, ¿necesitó contar con licencia o permiso por estado de maternidad? (Leer alternativas, respuesta única)

- ◯ Sí, y la licencia fue otorgada
- ◯ Sí, pero la licencia no fue otorgada
- ◯ No necesitó licencia o permiso por maternidad

38 ¿Por cuánto tiempo le otorgaron la licencia por maternidad?

- ◯ 90 días o más
- ◯ De 45 a 90 días
- ◯ Menos de 45 días
- ◯ No le otorgaron licencia por maternidad

39 ¿Recibió usted el subsidio por maternidad otorgado por ESSALUD?

- ◯ Sí
- ◯ No

40 Después de dar a luz o culminada su licencia por maternidad, describa su retorno al puesto de trabajo: (Leer alternativas, respuesta única)

- ◯ Retornó a su mismo trabajo
- ◯ Fue despedida por sus empleadores
- ◯ Tuvo que renunciar
- ◯ Tuvo que cambiar a otro trabajo

SECCIÓN 5 - EXPERIENCIA Y RESPONSABILIDADES LABORALES

41 ¿Cuáles de las siguientes labores o actividades realiza en su trabajo principal como trabajadora del hogar? Indique todas las que realice. (Leer alternativas, respuesta múltiple)

- ☐ Limpieza/Mantenimiento/cuidado del hogar
- ☐ Cocina/ayudante de cocina
- ☐ Lavado/planchado de ropa u otros similares
- ☐ Asistencia/cuidado de niñas/os y adolescentes
- ☐ Asistencia/cuidado de personas adultas mayores y/o personas enfermas
- ☐ Asistencia/cuidado de personas con discapacidad y/o personas dependientes del hogar
- ☐ Asistencia/cuidado de mascotas domésticas
- ☐ Jardinería
- ☐ Compras del hogar/mandados
- ☐ Otros ______

---

42 Además de su principal trabajo remunerado, ¿tiene otro(s) empleo(s) remunerado(s)? (Leer alternativas, respuesta única)

- ◯ No tiene otro trabajo remunerado
- ◯ Sí, frecuentemente
- ◯ Sí, ocasionalmente

---

43 ¿En qué actividades se desempeña en su(s) otro(s) trabajo(s) remunerado(s)? (Leer alternativas, respuesta múltiple)

- ◯ Trabajadora del hogar
- ◯ Comercio
- ◯ Limpieza
- ◯ Cocina
- ◯ Otros servicios
- ◯ Otras actividades

---

44 ¿Cuántas horas a la semana en promedio dedica a otro(s) trabajo(s) distintos de su principal trabajo remunerado?

Indicar número de horas a la semana ______

En los últimos 12 meses, ¿ha cambiado su trabajo en alguno de los siguientes aspectos? (Leer alternativas, respuesta única)

	Ha aumentado mucho	Ha aumentado algo	No ha cambiado	Se ha reducido algo	Se ha reducido mucho
45.1 El número de horas que trabaja a la semana	○	○	○	○	○
45.2 Su salario o ingresos	○	○	○	○	○
45.3 Sus tareas y deberes	○	○	○	○	○

SECCIÓN 5 - EXPERIENCIA Y RESPONSABILIDADES LABORALES

46 ¿Usted laboró antes como trabajadora del hogar?

- ◯ Sí
- ◯ No

47 Tomando en cuenta su experiencia anterior como trabajadora del hogar (su centro de labores anterior al actual), ¿cuánto tiempo laboró en dicho lugar?

- ◯ Un año o menos
- ◯ De 1 a 2 años
- ◯ De 2 a 3 años
- ◯ Más de 3 años
- ◯ No recuerda

48 Durante toda su vida, ¿cuántos años en total ha laborado como trabajadora del hogar?

Años: ______ Meses: ______

49 En promedio, ¿cuánto gasta diariamente en transporte para desplazarse al trabajo y viceversa para su trabajo principal? (Respuesta espontánea, única)

______ ______

50 En total, ¿cuánto tiempo al día dedica habitualmente a desplazarse desde casa al trabajo y vuelta a casa? (Ejm. Horas 2 y Minutos 30)

Horas: ______ y Minutos: ______

51 ¿Durante los últimos 12 meses (o desde que empezó a trabajar como trabajadora del hogar) ha participado de actividades de formación/capacitación para mejorar sus habilidades como trabajadora del hogar? (Respuesta múltiple)

- ☐ Formación pagada o proporcionada por empleador/a
- ☐ Formación pagada por Ud. misma
- ☐ Formación gratuita impartida por el sindicato
- ☐ Formación gratuita impartida por alguna organización del Estado
- ☐ Formación gratuita impartida por alguna organización civil
- ☐ Otro ______
- ☐ Ningún tipo de formación

52 En general, ¿qué curso/s de formación/capacitación usted siguió en los últimos 12 meses? (indicar cantidad de horas totales) (Respuesta espontánea, múltiple)

- ☐ Cocina / gastronomía / repostería ______
- ☐ Primeros auxilios / enfermería ______
- ☐ Jardinería / paisajismo ______
- ☐ Limpieza / mantenimiento del hogar ______
- ☐ Conducción de vehículos ______
- ☐ Otro ______ ______
- ☐ Ningún curso de formación

SECCIÓN 6 - ASPECTOS FAMILIARES Y PERSONALES

53 ¿Es usted la persona que más contribuye a los ingresos de su hogar?

- ◯ Sí
- ◯ No
- ◯ Todos por igual

54 ¿Qué número de miembros tiene su hogar (donde vive su familia), incluyéndose usted?

____

55 ¿Cuánto es el ingreso mensual familiar aproximado? (Considerando sus ingresos como trabajadora del hogar y otros ingresos en el hogar) (Leer alternativas, respuesta única) *Encuestador/a: recuerde que esta pregunta se refiere al ingreso total entre todos los integrantes de la familia que aportan dinero en el hogar. Tome en cuenta que esta pregunta es para quienes tienen más de un aportante de ingresos [adicionales a la TdH]

______ {ingreso_m_monto}

56 En general, ¿qué tan satisfecha está con las condiciones de trabajo de su principal trabajo remunerado? (Leer alternativas, respuesta única)

- ◯ Muy satisfecha
- ◯ Satisfecha
- ◯ Ni satisfecha ni insatisfecha
- ◯ Insatisfecha
- ◯ Muy insatisfecha

57 ¿Qué tan preocupada está por lo difícil que sería encontrar otro trabajo en caso usted quedara desempleada? (Leer alternativas, respuesta única)

- ◯ Nada preocupada
- ◯ Poco preocupada
- ◯ Más o menos preocupada
- ◯ Algo preocupada
- ◯ Muy preocupada

58 Suponiendo que pudiera elegir libremente sobre sus horas de trabajo y teniendo en cuenta la necesidad de ganarse la vida: ¿cuántas horas semanales preferiría trabajar actualmente? (Respuesta espontánea)

- ◯ El mismo número de horas que en la actualidad
- ◯ Una cantidad de horas diferente ______
- ◯ No sabe/No contesta

59 ¿Hasta qué edad le gustaría trabajar como trabajadora del hogar? (Respuesta espontánea)

- ◯ Lo más tarde posible / hasta que las energías lo permitan
- ◯ Una edad determinada ______
- ◯ No sabe/No contesta

SECCIÓN 7 - CONDICIONES DE SEGURIDAD LABORAL

En su ocupación principal y en una jornada laboral habitual, usted, ¿con qué frecuencia ...? (Leer alternativas, respuesta única)

		Siempre	Muchas veces	Algunas veces	Casi nunca	Nunca
60.1	¿Está expuesto a un nivel de ruido que le obliga a elevar el volumen de la voz para conversar con otra persona?	○	○	○	○	○
60.2	Aplica o manipula o está en contacto con sustancias químicas nocivas/tóxicas como: solventes, pesticidas (productos de jardinería), bencina, limpiadores desinfectantes (tipo ácido muriático, cloro (lejía), colorantes, pintura, entre otros	○	○	○	○	○
60.3	¿Respira sustancias químicas en forma de polvo, humos, aerosoles, vapores, gases? (No incluye el humo de tabaco)	○	○	○	○	○
60.4	Manipula o está en contacto con, animales (ejemplo: paseando o tratando con perros, gatos o la mascota de su empleador)	○	○	○	○	○
60.5	¿Está expuesta directamente a los rayos del sol o radiación por un periodo mínimo de 1 hora diaria?	○	○	○	○	○

61 ¿Su empleador le proporciona implementos o equipos de protección personal de acuerdo a las necesidades de las actividades que realiza? (por ejemplo: guantes, mascarillas, delantal, guardapolvo)

- ○ Sí
- ○ No

62 ¿Usa usted un uniforme de trabajo (brindado por el empleador o adquirido por cuenta propia) para realizar sus labores como trabajadora del hogar en el lugar donde trabaja? (Respuesta única, leer alternativas) *Encuestador/a: considere que uniforme hace referencia a "ropa de trabajo", "vestimenta laboral"; es decir: prenda de uso exclusivo para la jornada laboral. Ej: mandil, guardapolvo, etc.

- ○ Sí, se lo proporcionó su empleador
- ○ Sí, lo adquirió por su cuenta
- ○ No, porque su empleador no se lo ha proporcionado
- ○ No, porque no es necesario utilizarlo
- ○ Me dijeron que use uniforme. Pero, no quise.
- ○ Otro ______

63 ¿Usted usa los equipos de protección personal siempre que se requiere?

- ○ Sí
- ○ No

64 En los últimos 12 meses, ¿ha tenido algún accidente durante sus horas de trabajo?

- ○ No he tenido accidentes
- ○ Sí he tenido por lo menos un accidente

65	¿Qué tipo de accidente tuvo? (Leer alternativas, respuesta múltiple)	◯ Golpes con algún objeto<br>◯ Caídas<br>◯ Quemaduras<br>◯ Cortes, raspones o laceraciones<br>◯ Intoxicación por manipulación o inhalación de químicos<br>◯ Otro/s ______
66	Cuando usted tuvo el accidente, ¿su empleador brindó las facilidades para su atención médica, en caso esta haya sido necesaria?	◯ Sí<br>◯ No<br>◯ No fue necesaria la atención médica

SECCIÓN 8 - AUSENTISMO LABORAL Y ENFERMEDAD

67 Durante los últimos tres meses, ¿cuántos días en total ha faltado o ha tenido que dejar de trabajar debido a una baja por enfermedad o por motivos de salud? ______________________________

68 ¿Cuántos de estos días que ha faltado (por baja) fueron causados por...

accidentes de trabajo? ______
problemas de salud relacionados o empeorados por el trabajo (excluyendo los accidentes)? ______

69 Durante los últimos tres meses, ¿cuántos días ha trabajado enferma? ______________________________

SECCIÓN 9 - CARACTERÍSTICAS DEL EMPLEADOR

70	En su trabajo principal como trabajadora del hogar, ¿es su actual jefe/a directo un varón o una mujer?	◯ Varón<br>◯ Mujer
71	En su trabajo principal como trabajadora del hogar, ¿tiene usted algún vínculo familiar (sanguíneo o por afinidad) con su actual jefe/a directo?	◯ Sí ______<br>◯ No
72	¿Qué personas viven en la vivienda donde usted trabaja? (indique cantidad)	☐ Personas adultas (menores de 60 años) ______<br>☐ Personas adultas mayores (mayores de 60 años) ______<br>☐ Adolescentes de 15 a 18 años ______<br>☐ Niños y adolescentes de 5 a 15 años ______<br>☐ Niños menores a 5 años ______
73	¿Qué mascotas habitan en la vivienda donde usted trabaja? (indique cantidad)	☐ Perro(s) ______<br>☐ Gatos(s) ______<br>☐ Otro ______<br>☐ Ninguna mascota

En qué medida está de acuerdo o en desacuerdo con las siguientes afirmaciones: (Leer alternativas, respuesta única)

	Totalmente de acuerdo	Parcialmente de acuerdo	Ni de acuerdo ni en desacuerdo	Parcialmente en desacuerdo	Totalmente en desacuerdo
74.1 Su jefe/a la respeta	○	○	○	○	○
74.2 Su jefe/a le felicita y reconoce su buen trabajo	○	○	○	○	○
74.3 Su jefe/a sabe hacer que se trabaje en equipo	○	○	○	○	○
74.4 Su jefe/a le da su opinión sobre su desempeño laboral	○	○	○	○	○
74.5 Su jefe/a fomenta y apoya su formación	○	○	○	○	○

SECCIÓN 9 - CARACTERÍSTICAS DEL EMPLEADOR

75 ¿Su empleador/a le brinda alguno de los siguientes beneficios por fuera de su remuneración mensual? (Leer alternativas, múltiple)

- ☐ Ropa
- ☐ Dinero (propinas)
- ☐ Alimentación (desayuno, almuerzo y/o cena)
- ☐ Pago por tiempos extra
- ☐ Apoyo económico en emergencias (problemas económicos, emergencias médicas, etc)
- ☐ Víveres
- ☐ Gastos educativos de sus hijos
- ☐ Otros ______
- ☐ Ninguno

---

76 ¿Su empleador/a le paga a tiempo y de acuerdo con el calendario laboral?

- ◯ Sí
- ◯ No

---

77 Durante los últimos 12 meses ¿ha tenido algún problema de índole estrictamente laboral en el lugar donde trabaja? (Ej. Falta de pagos, vacaciones, beneficios sociales, hostigamiento, etc.)

- ◯ Sí
- ◯ No

---

78 ¿Reportó alguno de estos problemas laborales a la autoridad laboral (ej. SUNAFIL)?

- ◯ Sí
- ◯ No

---

79 ¿Cuál fue la razón para no denunciar estos problemas laborales? (Respuesta espontánea, múltiple)

- ◯ Tenía miedo de perder su trabajo
- ◯ Le preocupaba que las quejas podrían dañar la relación son su empleador/a
- ◯ Me preocupa que mi empleador brinde referencias negativas sobre mi trabajo
- ◯ Le preocupaba que le redujeran su salario o sus horas de trabajo
- ◯ Le daba miedo que su empleador/a se ponga violento/a
- ◯ No sabía cómo proceder / a dónde quejarse o denunciar
- ◯ Otra razón ______

Durante el último año, ¿ha sido sometida durante el transcurso de su trabajo a alguna de las siguientes situaciones? (Leer alternativas, respuesta única)

	Nunca	Casi nunca	Algunas veces	Casi siempre/	siempre
80.1 Ofensas verbales	◯	◯	◯	◯	◯
80.2 Amenazas/acoso/intimidación	◯	◯	◯	◯	◯
80.3 Comportamientos humillantes	◯	◯	◯	◯	◯
80.4 Violencia física	◯	◯	◯	◯	◯
80.5 Violencia por racismo o discriminación	◯	◯	◯	◯	◯
80.6 Acoso sexual	◯	◯	◯	◯	◯

SECCIÓN 9 - CARACTERÍSTICAS DEL EMPLEADOR

81	¿Reportó alguna de estas situaciones a alguna autoridad competente? (Ej. SUNAFIL, Policía Nacional, Centro de Emergencia Mujer, Línea 100, Ministerio Público, Poder Judicial, etc.)	◯ Sí<br>◯ No
82	¿Cuál fue la razón para no denunciar estas situaciones? (Respuesta espontánea, múltiple)	◯ Tenía miedo de perder su trabajo<br>◯ Le preocupaba que las quejas podrían dañar la relación son su empleador/a<br>◯ Me preocupa que mi empleador brinde referencias negativas sobre mi trabajo<br>◯ Le preocupaba que le redujeran su salario o sus horas de trabajo<br>◯ Le daba miedo que su empleador/a se ponga violento/a<br>◯ No sabía cómo proceder / a dónde quejarse o denunciar<br>◯ Otra razón ______

Comentarios/observaciones del encuestador (opcional)

____

ANITA: "Abordando los Desafíos y Limitaciones de las Políticas de Protección Social para las Trabajadoras del Hogar en el Perú"

Módulo 5 - Conocimientos y percepciones sobre protección social

SECCIÓN 1 - SOBRE LA LEY N° 31047

duracion ________________________________

1 ¿Ha escuchado sobre la ley 31047 sobre trabajadoras y trabajadores del hogar?
- ◯ Sí
- ◯ No

2 ¿A través de qué medio se enteró sobre esta ley sobre trabajadoras y trabajadores del hogar?
- ◯ A través de otra trabajadora del hogar
- ◯ A través de su sindicato u organización
- ◯ A través de la agencia de empleos
- ◯ A través de su empleador
- ◯ En la televisión
- ◯ En la radio
- ◯ En un periódico, semanario o revista
- ◯ En la red social Facebook
- ◯ En la red social Twitter
- ◯ En la red social TikTok
- ◯ En la red social Instagram
- ◯ En la red social WhatsApp
- ◯ De otra manera ______

3 Según usted, ¿qué derechos de las trabajadoras del hogar garantiza esta ley? (Respuesta múltiple)
- ☐ Derecho a mejor sueldo
- ☐ Derecho al cumplimiento de vacaciones
- ☐ Derecho a la salud
- ☐ Derecho a mejores condiciones de higiene en el trabajo
- ☐ Derecho a mejores condiciones de seguridad en el trabajo
- ☐ Derecho a un mejor y pertinente descanso
- ☐ Derecho a organizarse
- ☐ Derecho a reconocerse como una población con derechos

4 ¿Usted considera que esta Ley es útil o no para la mejora de sus condiciones laborales?
- ◯ Sí es útil
- ◯ No es útil
- ◯ No sabe / No responde

5 Respecto al cumplimiento de la Ley, ¿usted diría que...? (Leer alternativas, respuesta única)
- ◯ Es una Ley que se está cumpliendo
- ◯ Es una Ley que no se está cumpliendo
- ◯ Es una Ley que se cumple a medias
- ◯ Es una ley que nunca se va a cumplir

6 ¿En qué plazo cree usted que se empiece a cumplir totalmente esta Ley? (Leer alternativas, respuesta única)
- ◯ Es una Ley que se cumplirá en el corto plazo (un año o menos)
- ◯ Es una Ley que se cumplirá en el mediano plazo (más de un año, menos de cinco años)
- ◯ Es una Ley que se cumplirá en el largo plazo (cinco años a más)
- ◯ No sabe / No responde

7 ¿Qué institución o autoridad es la principal responsable para lograr el cumplimiento de esta ley? (Respuesta múltiple)

- ☐ La Presidenta de la República
- ☐ El Ministerio de Trabajo
- ☐ El Congreso de la República
- ☐ SUNAFIL
- ☐ Los sindicatos de trabajadoras del hogar
- ☐ Otras organizaciones de trabajadoras del hogar
- ☐ Las trabajadoras del hogar
- ☐ Los empleadores de trabajadoras del hogar
- ☐ Otro(s) ______

SECCIÓN 2 - SOBRE EL CONTRATO DE TRABAJO

8	¿Nos podría decir qué plazo tiene su contrato vigente? (Leer alternativas, respuesta única)	◯ Seis meses o menos<br>◯ Mayor a seis meses y menor a un año<br>◯ Un año o más<br>◯ Tiene plazo indeterminado<br>◯ No tiene contrato de trabajo<br>◯ No sabe / No responde
9	En caso cuente con contrato laboral, conoce si su empleador/a ha registrado su contrato en la página web del Ministerio de Trabajo y Promoción del Empleo?	◯ Sí, está registrado<br>◯ No, no está registrado<br>◯ No sabe / no está segura
10	¿Tiene usted alguno de estos tipos de contrato? (Leer alternativas, respuesta única)	◯ Contrato de suplencia<br>◯ Contrato de emergencia<br>◯ Contrato para servicio específico<br>◯ Contrato de temporada<br>◯ Contrato simple de trabajo doméstico<br>◯ Otro tipo de contrato específico ______<br>◯ No sabe / No responde
	¿Tiene algún cargo directivo en la organización a la que pertenece?	◯ Sí ______<br>◯ No

SECCIÓN 3 - SOBRE LA REMUNERACIÓN Y OTROS BENEFICIOS

11	A continuación, le formularemos algunas preguntas sobre algunas condiciones laborales importantes en su centro de labores.<br>¿Su empleador le descuenta por concepto de alimentación o alojamiento? (Leer alternativas, respuesta única)	◯ Le descuentan por concepto de alimentación<br>◯ Le descuentan por concepto de alojamiento<br>◯ Le descuentan por concepto de alimentación y alojamiento<br>◯ No le realizan ningún descuento y le brindan alimentación y/o alojamiento<br>◯ No toma alimentos ni se aloja en su centro de labores
12	Hace unos minutos me comentó que su empleador le paga por transferencia bancaria. ¿El pago mediante transferencia es realizado con su consentimiento o por acuerdo con su empleador?	◯ Le transfieren con su consentimiento o acuerdo entre ambas partes<br>◯ Le trasfieren sin su consentimiento o sin acuerdo entre ambas partes
13	¿Su empleador le extiende una boleta de pago por sus labores como trabajadora del hogar? (Leer alternativas, respuesta única)	◯ Sí<br>◯ No
14	¿Con qué frecuencia su empleador la extiende boletas de pago? (Leer alternativas, respuesta única)	◯ Cada quince días<br>◯ Cada mes<br>◯ Cada dos meses<br>◯ Irregularmente<br>◯ Otro ______
15	¿A cuánto ascendieron los montos de las gratificaciones que recibió el año 2022? (Leer alternativas, respuesta única)	◯ A un sueldo mensual completo en julio y otro sueldo más en diciembre<br>◯ A una fracción del sueldo mensual en julio y otra fracción en diciembre<br>◯ A un sueldo mensual completo solamente en julio / solamente en diciembre<br>◯ A una fracción del sueldo mensual solamente en julio / solamente en diciembre<br>◯ No recibí gratificación alguna

SECCIÓN 4 - SOBRE LA JORNADA LABORAL Y DESCANSOS

16	En sus horas libres, ¿usted realiza actividades fuera de su centro de labores o permanece en su centro de labores?	◯ Realiza actividades fuera de su centro de labores<br>◯ Realiza actividades en su centro de labores<br>◯ Realiza actividades en su centro de labores y fuera de él<br>◯ No tiene horas libres nunca<br>◯ Otro ______
17	¿Qué actividades realiza durante sus horas libres? (Respuesta espontánea, múltiple)	☐ Sale a visitar a su familia o amistades<br>☐ Sale de paseo<br>☐ Hace deporte<br>☐ Usa Internet (para navegar, comunicarse, ver videos, etc.)<br>☐ Estudia<br>☐ Realiza alguna actividad económica o productiva<br>☐ Descansa / reposa / duerme<br>☐ Otra actividad ______
18	¿Cuántos días de vacaciones cree usted que le corresponde por un año de trabajo? (Espontánea)	________________________________

SECCIÓN 5 - SOBRE EL VÍNCULO LABORAL

19 En los últimos 12 meses, ¿usted renunció a algún centro de labores donde se desempeñaba como trabajadora del hogar?

- ◯ Sí
- ◯ No

20 Al renunciar, ¿usted tuvo algunos de estos inconvenientes?

- ◯ Tuvo inconvenientes con el plazo de preaviso al empleador
- ◯ Tuvo inconvenientes con el pago CTS
- ◯ Tuvo inconvenientes con el pago de beneficios sociales
- ◯ No tuvo inconvenientes
- ◯ Otro ______

21 En los últimos 12 meses, ¿usted fue despedida de algún centro de labores donde se desempeñaba como trabajadora del hogar?

- ◯ Sí
- ◯ No

22 Al ser despedida, ¿qué motivo o razón le dio su empleador para su despido? (Leer alternativas, respuesta única)

- ◯ Por no cumplir adecuadamente con sus labores
- ◯ Por problemas económicos de su empleador
- ◯ Por solicitar aumento de sueldo
- ◯ Por mutuo acuerdo
- ◯ Por solicitar beneficios de ley
- ◯ No dio ningún motivo
- ◯ Otro ______

23 Al culminar su relación laboral, sea por renuncia o despido ¿usted recibió liquidación?

- ◯ Sí
- ◯ No

24 En los últimos 12 meses, ¿usted fue colocada laboralmente por alguna agencia de empleo?

- ◯ Sí
- ◯ No

25 ¿La agencia de empleo le cobró algún importe por colocarla laboralmente? (Leer alternativas, respuesta única)

- ◯ Sí, un % menor al 30% de su primer sueldo.
- ◯ Sí, el aproximadamente el 30% de su primer sueldo.
- ◯ Sí, el aproximadamente el 50% de su primer sueldo.
- ◯ Sí, el 100% de su primer sueldo.
- ◯ Otro monto ______
- ◯ No

SECCIÓN 6 - SOBRE LA SINDICALIZACIÓN

26 ¿Con qué frecuencia participa de actividades, reuniones de su organización de trabajadoras del hogar? (Espontánea)

- ◯ Semanal
- ◯ Quincenal
- ◯ Mensual
- ◯ Trimestral
- ◯ Anual
- ◯ Nunca

PARA EL(LA) ENTREVISTADOR(A):

La(s) asociación(es) marcada(s) en la pregunta 10 de la encuesta 0 es(son):
nota: Toma esta información como referencia para preguntar por el tiempo de pertenencia en cada una de las siguientes asociaciones.

[pertenece_asoc_esp]

---

27 ¿Desde qué tiempo es parte de dicha organización(es)?

Organización 1 Número de meses: ______ Número de años: ______
Organización 2 Número de meses: ______ Número de años: ______
Organización 3 Número de meses: ______ Número de años: ______
Organización 4 Número de meses: ______ Número de años: ______
Organización 5 Número de meses: ______ Número de años: ______
Organización 6 Número de meses: ______ Número de años: ______
Organización 7 Número de meses: ______ Número de años: ______
Organización 8 Número de meses: ______ Número de años: ______
Organización 9 Número de meses: ______ Número de años: ______
Organización 10 Número de meses: ______ Número de años: ______

---

28 Si pertenece a alguna organización, su empleador/a le dificulta directa/indirectamente su participación en las reuniones de la organización?

- ◯ Sí
- ◯ No

---

29 Usted nos indicó que se encuentra afiliada a, por lo menos, una asociación, organización, sindicato o federación. Como integrante, ¿paga alguna cuota mensual/anual? Detallar

- ◯ Sí ______
- ◯ No
- ◯ No sabe

---

30 Hace un momento mencionó que ocupa un cargo directivo en su sindicato/federación, ¿goza de algún beneficio por ocupar dicho cargo? Especificar

- ◯ Si, licencia sindical de 30 días por año calendario.
- ◯ Si, otro ______
- ◯ Ninguno
- ◯ No sabe

---

31 Dado que nos indicó anteriormente que no se encuentra afiliada a ningún sindicato/federación, ¿se animaría a hacerlo?

- ◯ Sí
- ◯ No

---

32 ¿Cuáles son sus razones para estar afiliada a un sindicato/federación? (Respuesta espontánea, múltiple)

- ◯ Por recomendación de personas cercanas
- ◯ Para gozar de beneficios sociales
- ◯ Para percibir una mejor remuneración
- ◯ Para tener estabilidad laboral
- ◯ Para relacionarme con mis colegas/pares
- ◯ Para tener respaldo legal
- ◯ Otro ______

33	¿Cuáles son sus razones para no estar afiliada a un sindicato/federación? (Respuesta espontánea, múltiple)	◯ Por recomendación de personas cercanas<br>◯ Porque no obtengo ningún beneficio<br>◯ Porque no lo considero necesario<br>◯ Porque cobran una cuota mensual/anual<br>◯ Porque me generaría problemas con mi empleador<br>◯ Porque no sé para qué sirve/de qué se trata<br>◯ Porque no sé cómo afiliarme<br>◯ Otro ______
34	¿Conoce si en su localidad hay algún sindicato?	◯ Sí<br>◯ No

SECCIÓN 7 - SOPORTE SOCIAL Y DISCRIMINACIÓN

A continuación, voy a leer una lista de enunciados acerca de su círculo social más cercano (afectivo, familiar, amical), a los que le pido responda de acuerdo a la siguiente escala: totalmente en desacuerdo, en desacuerdo, ni de acuerdo ni en desacuerdo, de acuerdo y totalmente de acuerdo (Leer alternativas, respuesta única)

	Totalmente en desacuerdo	En desacuerdo	Ni de acuerdo ni en desacuerdo	De acuerdo	Totalmente de acuerdo
35.1 Hay una persona en especial que está cerca cuando yo estoy en necesidad	○	○	○	○	○
35.2 Hay una persona en especial con la cual yo puedo compartir mis alegrías y mis penas (lamentos)	○	○	○	○	○
35.3 Mi familia realmente trata de ayudarme	○	○	○	○	○
35.4 Yo recibo la ayuda emocional y el apoyo que necesito de mi familia	○	○	○	○	○
35.5 Yo tengo una persona en especial la cual es verdaderamente una fuente de consuelo para mí	○	○	○	○	○
35.6 Mis amistades realmente tratan de ayudarme	○	○	○	○	○
35.7 Yo puedo contar con mis amistades cuando las cosas salen mal	○	○	○	○	○
35.8 Yo puedo hablar de mis problemas con mi familia	○	○	○	○	○
35.9 Yo tengo amistades con las cuales yo puedo compartir mis alegrías y mis penas (lamentos)	○	○	○	○	○
35.10 Hay una persona en especial en mi vida a quien le importa mis sentimientos	○	○	○	○	○
35.11 Mi familia está dispuesta a ayudarme a hacer decisiones	○	○	○	○	○
35.12 Yo puedo hablar de mis problemas con mis amistades	○	○	○	○	○

36 Hace un momento usted mencionó que está de acuerdo con la frase de que hay una "persona especial" en su vida en determinadas situaciones. En relación con su respuesta a la sección anterior, ¿qué relación tiene con "esa persona especial o personas? (Espontánea, múltiple)

- ☐ Pareja/Conviviente
- ☐ Enamorado/a
- ☐ Amigo/a
- ☐ Profesional de confianza (médico/a, consejero/a, terapeuta, pastor/a, sacerdote, profesor)
- ☐ Compañera trabajadora
- ☐ Empleador/a
- ☐ Otro familiar
- ☐ Otros ______

En su vida diaria, ¿con qué frecuencia le ha sucedido alguna de las siguientes situaciones? (Leer alternativas, respuesta única)

	Casi todos los días	Al menos una vez por semana	Algunas veces al mes	Algunas veces al año	Menos de una vez al año	Nunca
37.1 Es tratada con menos cortesía que otras personas	◯	◯	◯	◯	◯	◯
37.2 Es tratada con menos respeto que otras personas	◯	◯	◯	◯	◯	◯
37.3 Recibe un peor servicio que otras personas en un restaurante o en una tienda	◯	◯	◯	◯	◯	◯
37.4 La gente actúa como si pensaran que usted no es inteligente	◯	◯	◯	◯	◯	◯
37.5 La gente actúa como si le tuvieran miedo	◯	◯	◯	◯	◯	◯
37.6 La gente actúa como si pensaran que usted deshonesta	◯	◯	◯	◯	◯	◯
37.7 La gente actúa como si fueran mejores que usted	◯	◯	◯	◯	◯	◯
37.8 La gente le pone sobrenombres o le insultan	◯	◯	◯	◯	◯	◯
37.9 Es amenazada o acosada	◯	◯	◯	◯	◯	◯

SECCIÓN 7 - SOPORTE SOCIAL Y DISCRIMINACIÓN

38	¿Cuál cree que es la razón principal para estas experiencias? (Respuesta espontánea)	◯ Su nacionalidad<br>◯ Su género<br>◯ Su raza o color de piel<br>◯ Su edad<br>◯ Su religión<br>◯ Su estatura<br>◯ Su peso<br>◯ Otro aspecto de tu apariencia física<br>◯ Su orientación sexual<br>◯ Su nivel de educación o nivel de ingreso<br>◯ Una discapacidad física<br>◯ Otro ______
39	Usted, en alguna oportunidad, ¿se ha sentido discriminada por su empleador?	◯ Sí<br>◯ No
40	En los últimos 12 meses, ¿en algún momento fue obligada por su empleador a usar mandiles, delantales u otro distintivo que la identifique como trabajadora del hogar en espacios o establecimientos públicos (como parques, plazas, playas, restaurantes, hoteles, locales comerciales, clubes sociales y otros similares)? *Encuestador/a: En caso sea necesario, especificar el término "mandil" como prenda que cuelga del cuello y es atada a la cintura que se usa durante la jornada laboral	◯ Sí<br>◯ No
41	¿Qué consecuencias negativas le trajo llevar obligatoriamente esa vestimenta? (Leer alternativas, respuesta múltiple)	◯ Discriminación en espacios o establecimientos públicos<br>◯ Violencia física<br>◯ Violencia psicológica<br>◯ Violencia sexual (incluye acoso)<br>◯ Otro(s) ______<br>◯ No ha tenido consecuencias negativas

SECCIÓN 8 - SOBRE LA SEGURIDAD SOCIAL Y SEGURIDAD PERSONAL

42 Hace algunos minutos, usted mencionó que está afiliada a un sistema previsional, o sea de pensiones. ¿Su empleador retiene el aporte correspondiente para el sistema de pensiones elegido por usted?

- ◯ Sí
- ◯ No
- ◯ No sabe / No responde

---

43 Usted, en su condición de persona extranjera, ¿se ha visto alguna vez afectada por agencias de empleadores de trata laboral?

- ◯ Sí
- ◯ No
- ◯ No sabe / No responde

SECCIÓN 9 - CONOCIMIENTO SOBRE PROGRAMAS DE PROTECCIÓN SOCIAL Y SU USO

44 De la siguiente lista de programas de protección o asistencia social, ¿cuáles conoce o ha escuchado mencionar alguna vez? (Múltiple, leer alternativas)

- ☐ CUNAMÁS: Cuidado diurno que incluye juego y nutrición a niñas y niños menores de 3 años (zonas rurales y urbanas) y acompañamiento a familias (incluye gestantes)
- ☐ JUNTOS: Programa de apoyo monetario directo a los hogares más pobres con gestantes, niñas, niños y adolescentes
- ☐ QALIWARMA:- Programa de alimentación escolar (inicial y primaria en escuelas públicas)
- ☐ PENSIÓN 65: Programa de apoyo a adultos mayores
- ☐ Pensión del Programa CONTIGO para discapacidad severa y situación de pobreza
- ☐ Techo Propio
- ☐ BECA 18: Becas integrales para carreras de educación superior
- ☐ Otros ______

---

45 ¿Ha hecho uso de algún programa de protección o asistencia social durante el último año?

- ◯ Sí ______
- ◯ No

---

46 ¿Recibió usted algún bono estatal durante la pandemia del COVID-19, como Yanapay (bono de 350 soles a personas en pobreza y extrema pobreza), Bono 600 (subsidio para trabajadores del sector público), Bono 210 (bono para trabajadores formales), entre otros? *Encuestador/a: En caso sea necesario, especificar el término "mandil" como prenda que cuelga del cuello y es atada a la cintura que se usa durante la jornada laboral

- ◯ Sí ______
- ◯ No
- ◯ No recuerdo

Comentarios/observaciones del encuestador (opcional)

____

Hora de término de la segunda ronda

____
