# Supplementary material for: Working conditions and labour rights coverage of women domestic workers in Peru: a respondent-driven sampling study
Source: BMC Public Health. 2026 Jul 14;26:2877. doi: 10.1186/s12889-026-28527-2 (PMC13647820; doi:10.1186/s12889-026-28527-2)
Supplement: Supplementary file 2 [file 12889_2026_28527_MOESM2_ESM.pdf]

Anita

Abordamos la protección social y el acceso a la salud para trabajadoras del hogar en Perú

VALE DE INCENTIVO

Número de cupón:

____

Lima Sur:
Local UPCH, Armendariz 497, Miraflores 15074

Lima Norte:
Local UPCH, Av. Honorio Delgado 430, San Martín de Porres 15102

Lima Centro:
Jr. Héroes de Tarapacá 132 Lima Cercado (Cuadra 1 de la Av. Brasil)

Los días lunes no se tomará encuesta debido a temas de logística

Anita

Abordamos la protección social y el acceso a la salud para trabajadoras del hogar en Perú

CUPÓN DE PARTICIPACIÓN

de la encuesta para trabajadoras del hogar del Proyecto ANITA

Número de cupón:

____

Dirección:

Lima Sur: Local UPCH, Armendariz 497, Miraflores 15074
Día y hora: Viernes: □; Domingo: □ ; de ____ hasta ____ horas del día

Lima Norte: Local UPCH, Av. Honorio Delgado 430, San Martín de Porres 15102
Día y hora: Martes: □ **;** Domingo □ ; de ____ hasta ____ horas del día

Lima Centro: Jr. Héroes de Tarapacá 132 Lima Cercado (Cuadra 1 de la Av. Brasil)
Día y hora: Miércoles □; de ____ hasta ____ horas del día

Los días lunes no se tomará encuesta

Recibo de incentivos :

Recibirán incentivos por cada persona que reclute que cumpla los requisitos y se inscriba en la encuesta.

No se pagarán incentivos en las siguientes condiciones:

- **Si ha pasado la fecha de caducidad (30 días a partir de la entrega)**
- **Si el cupón ha sido adulterado/ en deterioro**
- **Si la referida no cumple los criterios de elegibilidad**
- **Si la referida ya ha participado en la encuesta**

Cupón de participación:

Este cupón le permite participar en la encuesta si cumple los criterios de selección y si completa todos los pasos de la encuesta.

Este cupón no se aceptará por las siguientes razones:

- **Si ha pasado la fecha de caducidad (30 días a partir de la entrega)**
- **Si el cupón ha sido adulterado / en deterioro**
- **Si no cumples los criterios de selección**
- **Si ha participado en la encuesta anteriormente**

Dudas y/o consultas

En caso de dudas y/o consultas puede comunicarse con Guillermo Almeida (cel: 941493235)
