# Supplementary material for: Working conditions and labour rights coverage of women domestic workers in Peru: a respondent-driven sampling study
Source: BMC Public Health. 2026 Jul 14;26:2877. doi: 10.1186/s12889-026-28527-2 (PMC13647820; doi:10.1186/s12889-026-28527-2)
Supplement: Supplementary file 3 [file 12889_2026_28527_MOESM3_ESM.docx]

**Supplementary material 3**

**Open-ended responses for other reasons for accessing or not labour rights benefits**

| **Open-ended text** | **frequency** |
| --- | --- |
| **Other reasons for having a written legal contract** |  |
| to have proof that it's working | 1 |
| to ensure that work schedules and wages are followed | 1 |
| **Other reasons for not having a written legal contract** |  |
| unclear | 1 |
| Disregard | 1 |
| Disregard | 2 |
| Employer refuses to recognize benefits | 1 |
| Don't think she needs it because employer offers all the benefits | 1 |
| She does not consider it necessary | 1 |
| Don't know | 1 |
| by the area where I work (Outside Lima Centre) | 1 |
| Because at first I was on a trial period, and then I would be offered a contract | 1 |
| Because the schedule is flexible | 1 |
| Because is a person who I know | 1 |
| Because is not a company | 1 |
| Because they did not talk about it | 1 |
| Because is a person who I know and there is trust | 1 |
| Because I don't work full time | 5 |
| Because someone else recommend me to work there | 1 |
| Because I work part time | 1 |
| They just asked if I want to work there | 1 |
| Because she work only 3 days a week in a household and then in another | 1 |
| **Other reasons for having retirement pension** |  |
| At her previous job, which wasn't like DWs, she enrolled in the AFP. Now she doesn't know if her current employer, like DW, deducts contributions from her pay and makes contributions to the AFP. | 1 |
| During the COVID pandemic she was enrolled to it ,she doesn't know how | 1 |
| My previous employer enrolled me in the plan; I had a contract there | 1 |
| I have monthly income | 1 |
| I'm in a payroll | 1 |
| I'm a widow, my husband enrolled me | 1 |
| **Other reasons for not having retirement pension** |  |
| I guess because of lack of contract | 1 |
| I believe that my current job does not meet the requirements for me to be enrolled in an AFP or ONP. | 1 |
| doesn't know how to sign up | 1 |
| is completely unfamiliar with the subject | 1 |
| is unfamiliar with the subject | 1 |
| Disregard | 1 |
| The deductions are high | 1 |
| for ESSALUD | 1 |
| It's part of her budget | 1 |
| It's her own fault; she'll do it in October | 1 |
| She is enrolled in an AFP, but she hasn't made any contributions to her AFP for the past 7 months. | 1 |
| Lack of knowledge | 1 |
| Lack of money for the deduction | 1 |
| Lack of information | 10 |
| My employer doesn't pay for it | 1 |
| I don't know what it is for | 1 |
| I don't know how to enroll, and I don't know what an AFP or an ONP is. | 1 |
| I don't know what an AFP or an ONP is. | 1 |
| I don't know | 1 |
| I don't know about it | 1 |
| does not have the financial resources to contribute | 1 |
| I don't want to enroll it | 1 |
| I don't have an insurance | 1 |
| I don't care about it | 1 |
| She hasn't had a job that would make her a member, nor does she know how to do it on her own | 1 |
| I didn't care too much about it | 1 |
| She didn't think much of it | 1 |
| I don't think is necesary | 1 |
| I don't know | 5 |
| I don't like it when they deduct money from my pay | 1 |
| I don't know | 6 |
| I don't know how to sign up | 1 |
| I don't know what it is for | 1 |
| I didn't know about this; she thinks it's only offered at large, private companies. | 1 |
| I didn't know I could enroll to AFP or ONP | 1 |
| Don't know | 6 |
| I don't know how to sign up | 1 |
| No one offer it to me | 1 |
| No one offer it to me, I didn't ask for it | 1 |
| I don't have a contract | 5 |
| I don't know about it | 2 |
| I don't have a contract and I don't have a payslip | 1 |
| I don't have money to contribute | 1 |
| I didn't know I could enroll to AFP or ONP | 1 |
| I don't know about it | 2 |
| Don't have contract | 7 |
| I don't have information, I don't know about the retirement system | 1 |
| I don't have stable job | 1 |
| I have never talked about it with my employer | 5 |
| No one offer it to me | 1 |
| Never offer | 1 |
| Lack of knowledge | 8 |
| Given the money they earn, they can't afford to pay for it | 1 |
| Lack of communication | 1 |
| I don't have labour benefits | 1 |
| I don't have a contract | 2 |
| Lack of formal job | 1 |
| Because I don't have a contract | 1 |
| Because I don't have an stable job | 1 |
| Scarce information | 1 |
| Lack of contract | 1 |
| because they haven't reached any agreement with the employee regarding that matter | 1 |
| because she feels she isn't working at a large company | 1 |
| because she isn't familiar with that type of insurance | 1 |
| because my previous employer didn't enroll me | 1 |
| Because I'm a migrant | 1 |
| Because I'm a minor | 1 |
| because that's for people who work in companies and factories, not for domestic workers | 1 |
| Because she was married and her husband was affiliated | 1 |
| I don't make much money | 1 |
| because I don't make much money and I can't afford it | 1 |
| My employer didn't offer me a formal contract | 1 |
| My employer never enroll me | 1 |
| My employer doesn't want to | 1 |
| My employer doesn't have information | 1 |
| I don't have an stable income | 1 |
| I don't have money to contribute | 1 |
| I'm not Peruvian | 1 |
| I'm not Peruvian and I don't know about it | 1 |
| I'm not in payroll | 6 |
| I don't know about it | 1 |
| because she isn't interested in contributing | 1 |
| because she has not been enrolled by her employer | 1 |
| because he hasn't signed any contract | 1 |
| because there is no employment contract | 1 |
| because I haven't spoken to my employer | 1 |
| because I haven't had a formal job | 1 |
| because I haven't worked at a company | 1 |
| Because they don't give him the benefit since he doesn't work for a company | 1 |
| because he hasn't attached any importance to it | 1 |
| because my employer hasn't offered it to me | 1 |
| because they don't pay me much | 1 |
| because my employer hasn't offered it | 1 |
| I don't have a contract | 11 |
| I don't have any insurance | 1 |
| because I don't have a job with a payslip | 1 |
| Because I don't have an stable job | 1 |
| Because she wasn't aware that she could enroll when she started working, and her employer didn't tell her anything. | 1 |
| because she doesn't receive benefits since she doesn't have a contract | 1 |
| Don't have a contract yet | 1 |
| because he doesn't have the contract, and therefore doesn't have those benefits | 1 |
| because he doesn't have insurance | 3 |
| because she doesn't have a steady job | 1 |
| because she doesn't have a contract | 5 |
| because she doesn't have a formal contract | 2 |
| I don't have an stable income | 1 |
| My job has no benefits | 1 |
| because she doesn't have a steady job with benefits | 1 |
| because she doesn't work for a company | 3 |
| I have never been in the payroll | 2 |
| I have never worked with a contract | 1 |
| No one told me | 1 |
| No one offer it | 1 |
| I have never had a contract | 1 |
| because, you know, it's not a legitimate company | 1 |
| Only companies do that | 1 |
| My employer didn't offer me a formal contract | 1 |
| My employer didn't offer that benefit | 1 |
| My job didn't offer it | 1 |
| Because I need to sign up | 1 |
| Part-time job | 1 |
| I don't have labor benefits | 1 |
| I don't have a formal job | 1 |
| I work certain days | 1 |
| I just got my resident ID card | 1 |
| si tuve afp pero retiré todo mi dinero en pandemia | 1 |
| no contract | 1 |
| Her employer says that since she is a DWs, she is not eligible, and she does not work for a company | 1 |
| The employer didn't mentioned it | 2 |
| He had ONP, but he lost all his money because he got married. He tried to file a claim to get his money back, but they never gave it to her | 1 |
| I don't have a employment contract | 1 |
| **Other reasons for having employer paid social security** |  |
| The husband affiliated her | 1 |
| Because is cheaper | 1 |
| because she had some money left over from a previous job many years ago, and she doesn't know how, but they assured her she would be covered for life | 1 |
| Because a percentage was set aside for his insurance when he was enrolled in an AFP; he is no longer enrolled in an AFP but still has the insurance. | 1 |
| **Other reasons for not having employer paid social security** |  |
| Lack of documentation and formal employment | 1 |
| Lack of information | 3 |
| Lack of time to learn how to do it | 1 |
| Lack of papers because she is an international migrant | 1 |
| Shee lost it when was 18, and she hasn't figured out how to get it back. | 1 |
| My employer has never mentioned to me that they are woing to enrol me in an insurance | 1 |
| She does have the economic means to cover it | 1 |
| She does not receive a payslip | 1 |
| It is not in her budget | 1 |
| Because she has not an estable job | 1 |
| She does not know how to be affiliated | 1 |
| Don't know | 7 |
| Employer did not offer it | 1 |
| She does not want to lose the SIS | 1 |
| I don't want to be deducted from my paycheck | 1 |
| I don't know how to get it | 1 |
| I don't know how to be affiliated | 1 |
| I didn't know I could be affiliated to ESSALUD | 1 |
| I don't have a contract | 3 |
| I don't have an stable job | 1 |
| I don't have a contract to be affiliated | 1 |
| I don't a proper job to be affiliated | 1 |
| I don't really care for my health | 1 |
| I don't know what ESSALUD is | 1 |
| I do not have a contract | 3 |
| I don't have information, I don't know | 1 |
| Lack of contract | 1 |
| Lack of money | 1 |
| Lack of a formal job | 1 |
| Because of the terrible service they provide | 1 |
| Because of financial issues and because I didn't have a contract | 1 |
| Because I'm not on the payroll | 1 |
| Because I don't have a contract | 2 |
| Beacuse I don't have a good job | 1 |
| Because when she started working, she already had her SIS, and she doesn't want them to take her SIS away all the time just because she's temporarily enrolled in Essalud. | 1 |
| Because my contract does not state that my employer provides me with that benefit | 1 |
| Because I am a migrant | 1 |
| because that's not for domestic workers, but for other sectors of the workforce | 1 |
| Because I don't make much money | 1 |
| Because she asked for it and they didn't give it to her; they told her they were going to do it, but they didn't | 1 |
| Because people told me the services are not good | 1 |
| Because my employer hasn't enrolled me | 1 |
| Because my employer doesn't know | 1 |
| Because I'm formally employed | 1 |
| Because I'm not on payroll | 3 |
| Because I don't have a job that allows me to enroll | 1 |
| Because I'm not paying for that | 1 |
| because he has not worked for companies | 1 |
| because I haven't been enrolled in health insurance through my job | 1 |
| because she doesn't know if he can do it on his own | 1 |
| because she doesn't know if she's going to stay in this job, and if that were the case, her boss would have to enroll her in the plan | 1 |
| lack of contract | 2 |
| Lack of contract to be in ESSALUD | 1 |
| I don't have a resident ID card | 1 |
| I don't have a contract | 1 |
| Beacuse I dont have a contract | 1 |
| Lack of contract | 5 |
| Lack of formal contract | 1 |
| Lack of job contract | 2 |
| Lack of a job contract | 1 |
| I don't have a fixed salary | 1 |
| I don't a job with labour benefits | 1 |
| I don't have a formal job | 2 |
| because she doesn't work with anyone who has offered to do so | 1 |
| I don't work for a company | 3 |
| I don't work full time | 1 |
| because she has never worked for a company | 1 |
| because she prefers to receive care at a private practice | 1 |
| because she got married and lost her health insurance and her pension | 1 |
| because they take so long to provide service | 1 |
| Beacuse you need to pay for it | 1 |
| because you have to contribute but don't have the financial means | 1 |
| because she has always liked being independent and receiving a full paycheck | 1 |
| Because I only have a verbal agreement | 1 |
| because her employer knows he has the SIS | 1 |
| Their daugthers' father just passed away. Before that she was treated in ESSALUD | 1 |
| I just got my resident ID card | 1 |
| Her employer hasn't given her any information about that; he just told her that once she has her ID card, she should get her SIS. | 1 |
| The employer didn't mentioned it | 2 |
| Contract | 1 |
| I have SIS and ESSALUD is far away | 1 |
| I don't have a job contract | 1 |
